# Engineering *Corynebacterium glutamicum* to produce the biogasoline isopentenol from plant biomass hydrolysates

**DOI:** 10.1186/s13068-019-1381-3

**Published:** 2019-02-27

**Authors:** Yusuke Sasaki, Thomas Eng, Robin A. Herbert, Jessica Trinh, Yan Chen, Alberto Rodriguez, John Gladden, Blake A. Simmons, Christopher J. Petzold, Aindrila Mukhopadhyay

**Affiliations:** 1 Graduate School of Advanced Integrated Studies in Human Survivability, Kyoto University, Sakyo-ku, Kyoto, Japan; 2 Japan Society for the Promotion of Science, Sakyo-ku, Kyoto, Japan; 3 Joint BioEnergy Institute, Emeryville, CA USA; 4 Biomass Science and Conversion Technology Department, Sandia National Laboratories, 7011 East Avenue, Livermore, CA 94550 USA; 5 Biological Systems and Engineering Division, Lawrence Berkeley National Laboratory, Berkeley, CA USA; 6 Environmental Genomics and Systems Biology Division, Lawrence Berkeley National Laboratory, Berkeley, CA USA

**Keywords:** *Corynebacterium glutamicum*, Sorghum, Hydrolysate, Ionic liquid pretreatment, Isopentenol, Isoprenol, 3-Methyl-3-buten-1-ol, HmgR

## Abstract

**Background:**

Many microbes used for the rapid discovery and development of metabolic pathways have sensitivities to final products and process reagents. Isopentenol (3-methyl-3-buten-1-ol), a biogasoline candidate, has an established heterologous gene pathway but is toxic to several microbial hosts. Reagents used in the pretreatment of plant biomass, such as ionic liquids, also inhibit growth of many host strains. We explored the use of *Corynebacterium glutamicum* as an alternative host to address these constraints.

**Results:**

We found *C. glutamicum* ATCC 13032 to be tolerant to both the final product, isopentenol, as well to three classes of ionic liquids. A heterologous mevalonate-based isopentenol pathway was engineered in *C. glutamicum*. Targeted proteomics for the heterologous pathway proteins indicated that the 3-hydroxy-3-methylglutaryl-coenzyme A reductase protein, HmgR, is a potential rate-limiting enzyme in this synthetic pathway. Isopentenol titers were improved from undetectable to 1.25 g/L by combining three approaches: media optimization; substitution of an NADH-dependent HmgR homolog from *Silicibacter pomeroyi*; and development of a *C. glutamicum ∆poxB ∆ldhA* host chassis.

**Conclusions:**

We describe the successful expression of a heterologous mevalonate-based pathway in the Gram-positive industrial microorganism, *C. glutamicum,* for the production of the biogasoline candidate, isopentenol. We identified critical genetic factors to harness the isopentenol pathway in *C. glutamicum*. Further media and cultivation optimization enabled isopentenol production from sorghum biomass hydrolysates.

**Electronic supplementary material:**

The online version of this article (10.1186/s13068-019-1381-3) contains supplementary material, which is available to authorized users.

## Background

Microbial hosts used for the rapid discovery and development of metabolic pathways can have drawbacks that limit their biotechnological applications beyond the laboratory scale. These disadvantages include inhibition from components of the growth media and toxicity from metabolic intermediates or final products [1]. Solutions to several industrially relevant parameters and host sensitivities have been described, especially in model microbes such as *Escherichia coli* or *Saccharomyces cerevisiae* [2, 3], and are increasingly considered important for scale-up in an industrial process. However, if genetic tractability does not limit the choice of host organism, it is reasonable that any microbe could be utilized as a host for the expression of a heterologous gene pathway. Rather than develop solutions for individual aspects of an industrially relevant process, we viewed the choice of a microbial host as a means to bypass product and process reagent toxicities that would be encountered in the industrial process.

*Corynebacterium glutamicum* is a biotechnologically relevant host that presents several ideal characteristics for scale-up, such as a rapid phenotypic adjustment in response to environmental changes (e.g., oxygen levels and substrate availability), which are major causes of performance losses in industrial scale bioreactors [4, 5]. Of particular relevance for renewable biofuel production is its capacity of simultaneously utilizing glucose and xylose, two major components of plant biomass hydrolysates [6, 7], as well as *p*-coumaric and ferulic acids as carbon sources [8, 9]. These factors have contributed to the development of *C. glutamicum* as a production host for many bioproducts [10–12]. As a broad category, terpene-based compounds represent a rich source of biofuels and product targets [13]. Production of terpene compounds has been explored in *C. glutamicum* but reported titers currently range from 0.2 to 23 mg/L for terpenes such as pinene and β-carotene, respectively [14]. Isopentenol (3-methyl-3-buten-1-ol) is a prominent example of a terpene compound that is desirable as both biogasoline as well as a platform chemical and has been developed in other microbial systems (e.g., *E. coli*) [15], but not in *C. glutamicum*.

Growth inhibitory effects of residual pretreatment reagents, used to release metabolizable carbon sources from plant biomass, is another factor to be considered in the use of renewable carbon sources. Ionic liquids (ILs) represent one such class of pretreatment reagents [16, 17] and have advantages over conventional pretreatments such as favorable sugar solubilization rates, less degradation of monosaccharides, and compatibility with downstream enzymatic processing [18]. ILs derived from 1-ethyl-3-methylimidazolium ([C_2_C_1_im]^+^) are known to be toxic against several eukaryotes [19] and Gram-negative bacteria [20]. New classes of ILs, such as cholinium ([Ch]^+^) derived ILs [21] and protic ILs (such as ethanolamine acetate [ETA][OAc] and diethanolamine acetate [DEOA][OAc]) are emerging as equally effective but less toxic reagents for this application [22]. In contrast to other microbial hosts [23, 24], the physiological response of *C. glutamicum* has not been explored in detail to any of these ILs that remain as residual components in the biomass hydrolysate.

**Fig. 1:**
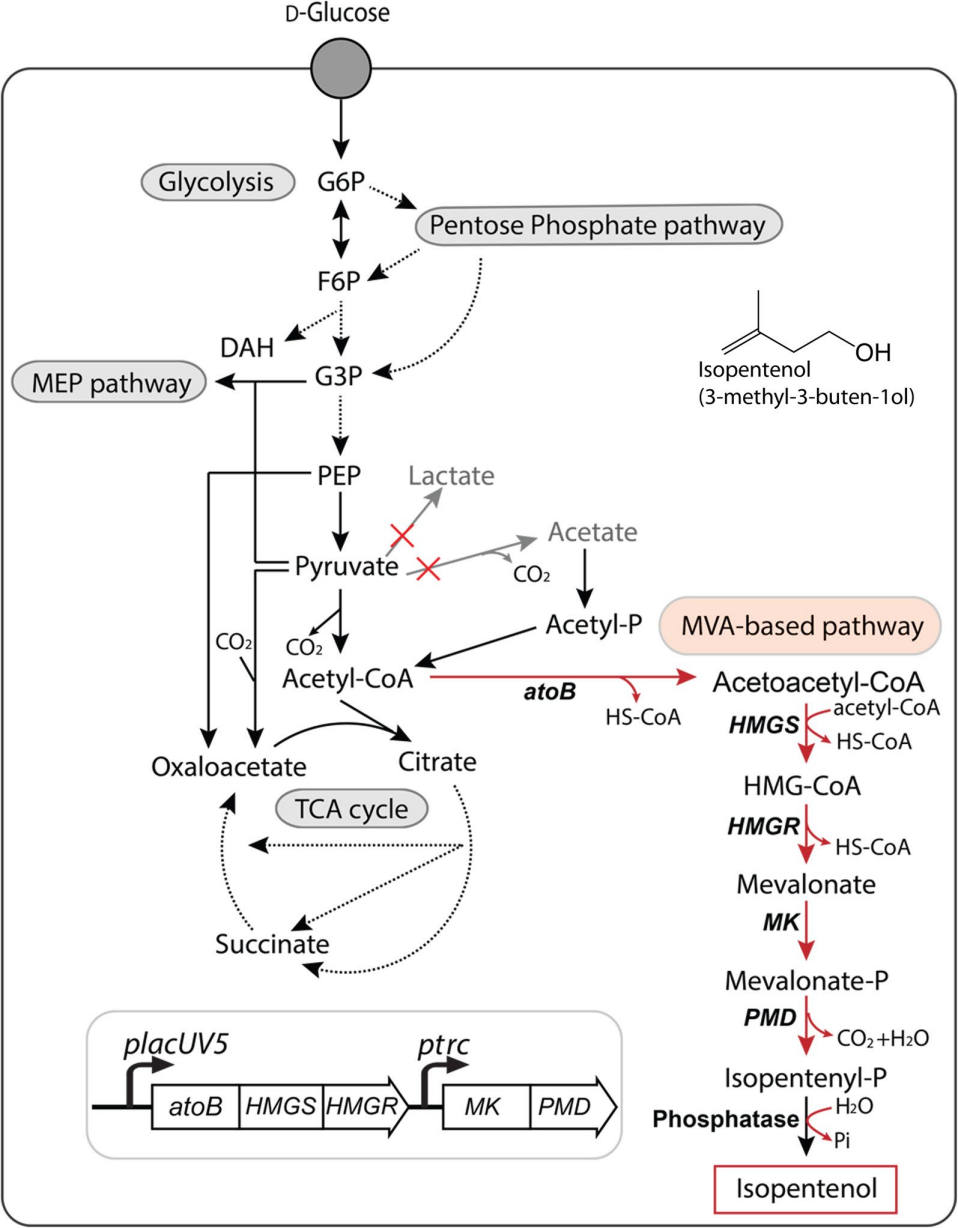
Global map of central metabolic pathways in *C. glutamicum* ATCC 13032 and the heterologous mevalonate-based pathway for isopentenol production. The mevalonate-based isopentenol biosynthesis pathway was described in [28]. Broken lines show intermediate pathways omitted in glycolysis and the tricarboxylic acid cycle. The disrupted pathways in this work are shown in grey and indicated with a red “X”. Relevant reactions are represented by the genes encoding their corresponding enzymes. Reactions involved in isopentenol biosynthesis are displayed with red arrows. Abbreviations: *atoB*, acetyl-CoA acetyltransferase; *HMGS*, hydroxymethylglutaryl-CoA synthase; *HMGR*, 3-hydroxy-3-methylglutaryl-CoA reductase; *MK*, mevalonate kinase; *PMD*, phosphomevalonate decarboxylase; MEP, methylerythritol-4-phosphate; MVA, mevalonate; G6P, glucose-6-phosphate; F6P, fructose-6-phosphate; DAH, dihydroxyacetone phosphate; G3P, glyceraldehyde-3-phosphate; PEP, phosphoenolpyruvic acid; HMG-CoA, 3-hydroxy-3-methyl-glutaryl-coenzyme A; HS-CoA, coenzyme A; Pi, inorganic phosphate

In this study, we explore the tolerance of *C. glutamicum* ATCC 13032 as a microbial platform for the heterologous mevalonate-based production pathway of isopentenol (Fig. 1). We characterized the innate tolerance of *C. glutamicum* to isopentenol, which is known to be toxic to other microbes [25], as well three classes of ILs. Using the proteomics, we identify a critical protein in the heterologous mevalonate pathway for isopentenol production. Furthermore, engineering the host strain background achieved isopentenol production in minimal defined media and IL extracted plant biomass hydrolysates in titers of 1.25 g/L and 1.1 g/L, respectively. These results highlight the potential of *C. glutamicum* as a sustainable production chassis to produce terpene-based biofuels and bioproducts.

## Results

### *C. glutamicum* is tolerant to three classes of ILs and exogenous isopentenol

**Fig. 2:**
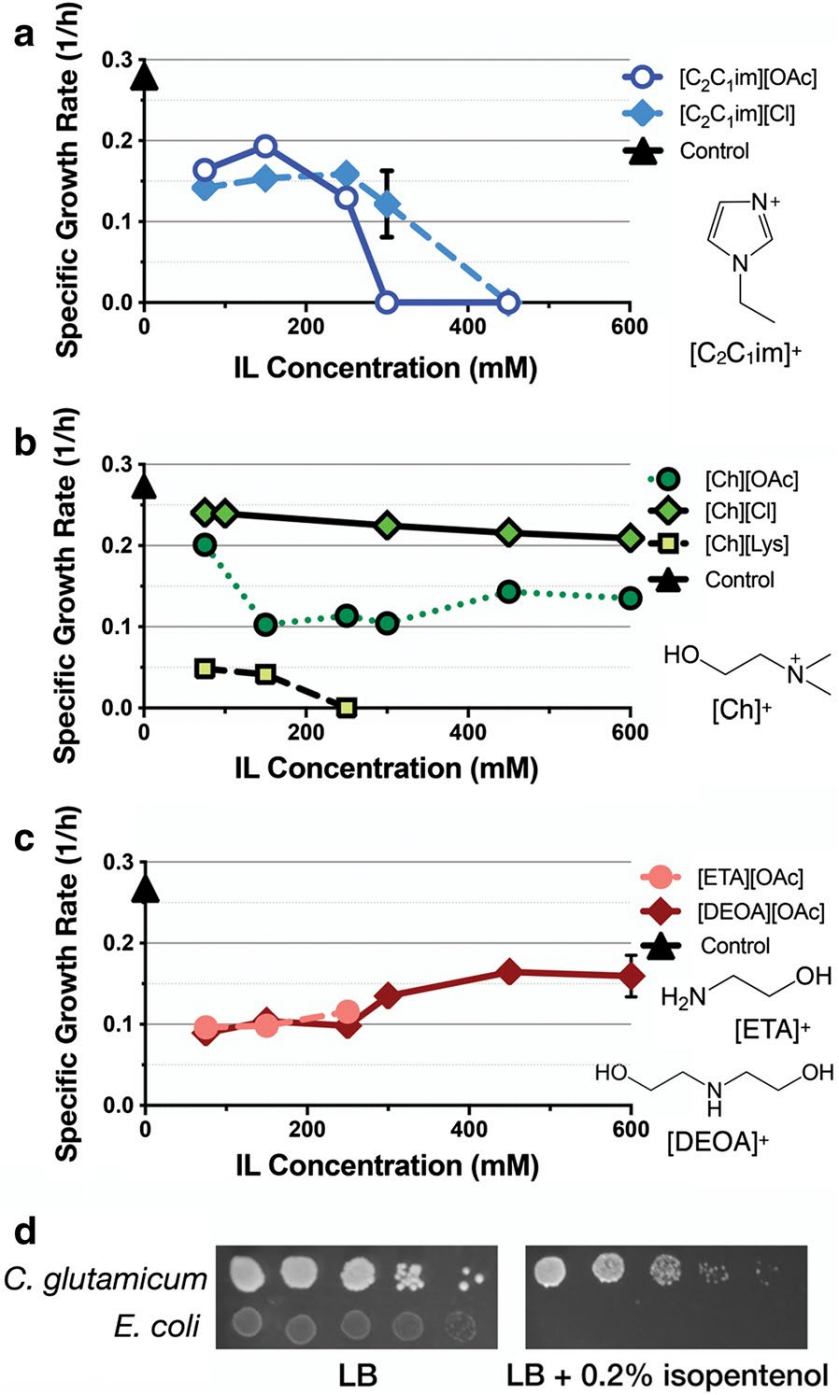
Assessment of *C. glutamicum* Resistance to Three Classes of ILs and Exogenous Isopentenol. **a**–**c** Growth assay for assessment of *C. glutamicum* resistance to ILs: *C. glutamicum* was cultured in CGXII minimal medium containing 4% d-glucose and the type and concentration of IL as indicated. Strains were cultivated in a 96 well microtiter plate. The growth rate in either control media or supplemented with exogenous IL as indicated (1/h) was calculated and plotted as a function of IL concentration. Data is the average of three independent biological replicates, and error bars indicate standard error. **d** Spot assay for assessment of *C. glutamicum* and *E. coli* resistance to isopentenol. Cells were serially diluted tenfold onto solid LB agar media with or without 0.2% (w/v) isopentenol as indicated and incubated at 30 °C. Error bars are not plotted when they are shorter than the symbol used on the graph. Photomicrographs were taken after plates were incubated for 2 days

We examined three broad IL classes: 1-ethyl-3-methyl imidazolium ([C_2_C_1_im]^+^) derived; cholinium ([Ch]^+^) derived; and protic: ethanolamine acetate [ETA][OAc] and diethanolamine acetate [DEOA][OAc]) in different cation/anion configurations, for toxicity against *C. glutamicum*. The specific growth rate of *C. glutamicum* in the absence of any IL was 0.28 doublings per hour. *C. glutamicum* grown in the presence of exogenous [C_2_C_1_im][OAc] or [C_2_C_1_im][Cl] indicated that this organism was tolerant to ~ 250 mM [C_2_C_1_im]^+^ (Fig. 2a). Its growth rate decreased fourfold and is a three to six fold increase in tolerance of [C_2_C_1_im]^+^ compared to wild-type *E. coli* (Additional file 1A). Of the cholinium based ILs, both [Ch][Lys] and [Ch][OAc] decreased growth rate by two to fourfold at 50 mM or higher. [Ch][Lys] was inhibitory at 250 mM, while [Ch][OAc] decreased growth rate but was not inhibitory at concentrations of 150 mM and above (Fig. 2b). [Ch][Cl] was nontoxic to *C. glutamicum*, and no growth defect was observed, even when supplemented to 600 mM (Fig. 2b). In contrast, *E. coli* was highly sensitive to [Ch][Lys], as it was inviable when [Ch][Lys] was added to the growth media at 40 mM (Additional file 1B). Ethanolamine- and diethanolamine-based ILs did not show a dosage dependent inhibition on *C. glutamicum* growth (Fig. 2c), suggesting that at tested concentrations these two representative protic ILs have no deleterious impact on *C. glutamicum* growth. As the concentration of ILs is below 100 mM in many commonly used hydrolysate preparations [26, 27], these results indicated that wild-type *C. glutamicum* was innately tolerant to most forms of the trace ILs remaining in extracted hydrolysates from biomass. No further optimization was required to grow *C. glutamicum* in the presence of additional ILs from these three classes.

To examine the tolerance of *C. glutamicum* to isopentenol and compare it against that in *E. coli* (known to show toxicity to exogenous isopentenol at a concentration of 0.2% (w/v) [25], we monitored and counted formation of colony forming units (CFUs) on agar plates. We confirmed that *E. coli* is inviable in the presence of 0.2% (w/v) isopentenol (Fig. 2d). In contrast, *C. glutamicum* is unperturbed at this concentration of isopentenol, as we observe a similar number of CFUs, albeit smaller and slower growing (Fig. 2d, compare the growth of LB plate to LB plus 0.2% (w/v) isopentenol plate). Wild-type *C. glutamicum* does not consume exogenous isopentenol in liquid cultures as detected by GC-FID after 24 or 48 h (Additional file 1C), where the decrease in isopentenol concentration was comparable to isopentenol evaporation in a side-by-side comparison (Additional file 1C). Isopentenol evaporation was dependent on the cultivation format and was more pronounced in a 24-well deep well plate format **(**Additional file 1D). These results indicated that *C. glutamicum* has a higher tolerance of exogenous isopentenol compared to *E. coli* and also that *C. glutamicum* does not consume this final product as a carbon source. Together, these results suggested that selecting *C. glutamicum* as the microbial chassis is justified due to its inherent tolerance for a suite of relevant biofuel-related molecules.

### Media, carbon and nitrogen levels dramatically impact isopentenol titers in *C. glutamicum*

Next, we expressed a heterologous mevalonate-based isopentenol biosynthesis pathway [28] in *C. glutamicum* (Fig. 1). Acetyl-coA is converted to mevalonate-phosphate by four enzymatic reactions, which in turn is decarboxylated to isopentenyl monophosphate (a promiscuous activity from *PMD*). Isopentenyl monophosphate is then spontaneously dephosphorylated by an endogenous phosphatase to yield isopentenol (Fig. 1). To identify the cultivation conditions for optimal production of isopentenol in *C. glutamicum,* we tested M9 and CGXII media, a *C. glutamicum* specific minimal media as well as LB (Lysogeny Broth), and conditions were determined based on previous studies for non-native metabolite production [29]. We detected robust isopentenol production in CGXII media, at ~ 250 mg/L after 24 h and ~ 380 mg/L after 48 h (Additional file 2A). However, no isopentenol was detected when the strain was grown in either M9 media or LB irrespective of the additional d-glucose supplementation from 1% (w/v) to 4% (w/v) (Additional file 2A, B). The d-glucose concentration was an apparent difference between the CGXII medium and the other media. These observations demonstrated that the starting d-glucose concentration alone is insufficient to induce isopentenol production in *C. glutamicum* and growth in M9 or LB inhibits the production of isopentenol. This result is in contrast to *E. coli*, where production of isopentenol (and terpenes in general) is typically robust under nutrient-rich conditions, but limited under minimal media conditions [13, 15].

**Fig. 3:**
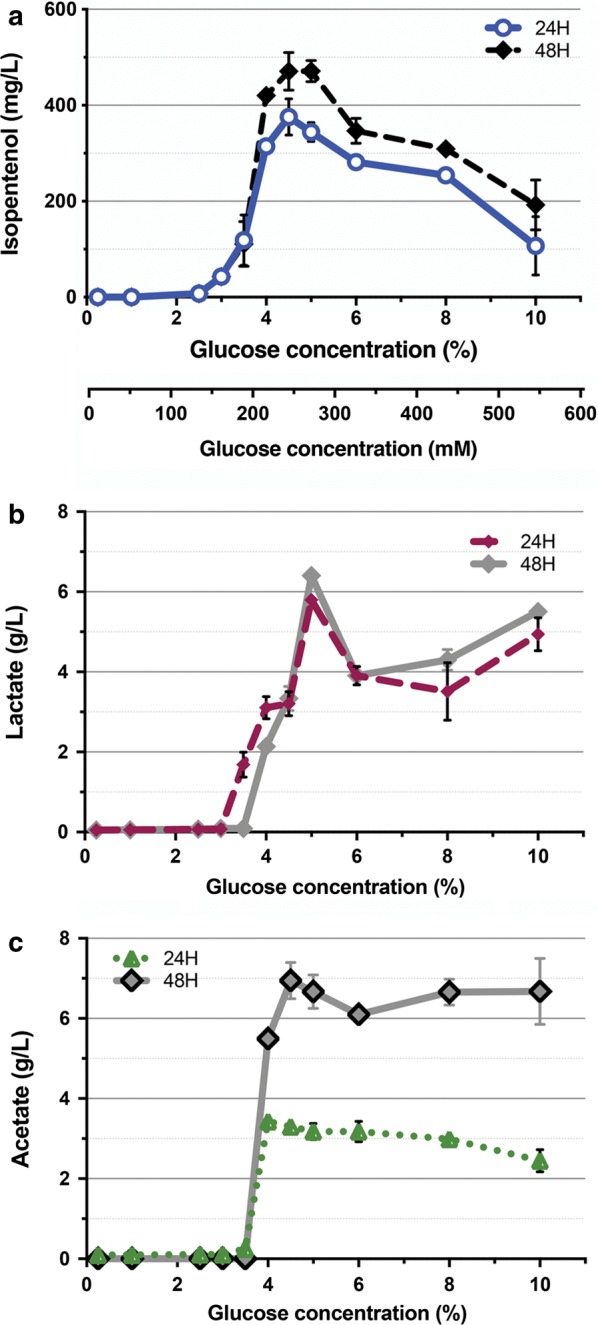
Analysis of Different d-Glucose Concentrations for Isopentenol Production in *C. glutamicum*. **a** Impact of different starting d-glucose concentrations on isopentenol production titer. *C. glutamicum* was cultivated for isopentenol production in CGXII medium amended with d-glucose concentration from 0.25% to 10% (w/v) (13.9 mM to 550 mM) in 24-well deep well plates. For clarity, both the mM and  % (w/v) d-glucose concentrations are indicated on the *x* axis. **b**, **c** Analysis of generated lactate (**b**) and acetate (**c**) titer during the isopentenol production at the 24 h and 48 h timepoints. Error bars are not plotted when they are shorter than the symbol used on the graph. For all experiments, data was generated from three independent biological replicates for each condition, and error bars indicate standard error

To examine the role of initial d-glucose concentration for production in CGXII media, we supplemented the medium with a range of starting d-glucose concentrations [0.25% to 10% (w/v)] and repeated the isopentenol production (Fig. 3a). No isopentenol was detectable in the strains grown with less than 2% of initial d-glucose (Fig. 3a). Above 4% of d-glucose, we detected close to ~ 450 mg/L of isopentenol titers after 48 h of induction (Fig. 3a). The isopentenol titers were comparable up to 8% of d-glucose but dropped threefold down to 100 mg/L at 10% of d-glucose (Fig. 3a). Under conditions where isopentenol production was detected, *C. glutamicum* strains first accumulated both lactate and acetate at the 24 h timepoint, and continued to accumulate additional acetate at the 48 h timepoint (Fig. 3b, c). The measured d-glucose concentration using HPLC indicated that it was completely consumed after 24 h in the strains grown with less than 4% d-glucose, but partially remained when they were grown with 4% or higher initial d-glucose concentration, even after 48 h (Additional file 3A). The isopentenol production strains also produced 1 g/L of succinate, which did not change appreciably over the range of initial d-glucose (Additional file 3B). While there were differences in OD_600_ values among these strains cultured in different initial d-glucose concentrations, there was no correlation between culture density with isopentenol titer, as *r*^2^ = 0.00053 (Additional file 3C). These results confirm that the starting d-glucose concentration can impact batch-mode production of isopentenol using *C. glutamicum*. A twofold change in the initial d-glucose concentration can have a 30-fold impact on isopentenol titer.

Next, we examined if the initial nitrogen concentration also influenced isopentenol titer. The nitrogen concentration in CGXII media was varied (the ratio of ammonium sulfate and urea were kept the same) and the d-glucose concentration was held constant at the standard 4% d-glucose (222 mM). There was a decrease in isopentenol titer as we varied the initial nitrogen concentration in either direction from the standard of 151 mM ammonium sulfate with 83.3 mM urea [30] (Additional file 3D). Historically, the carbon:nitrogen ratio (C:N ratio) is known to impact the detectable levels of metabolites in log phase cultures [31]. The C:N ratio in the published CGXII media is 2.8 (6 × 222 mM d-glucose/2 × 151 mM ammonium sulfate plus 2 × 83.3 mM urea). We combined the data for isopentenol production when we varied either nitrogen or d-glucose and re-plotted isopentenol production as a function of the C:N ratio (Additional file 3E). The optimal C:N ratio in CGXII media was between 2.8 and 4.3. The C:N ratio in M9 media is considerably higher at 17.8 (6 × 55.5 mM d-glucose/18.7 mM ammonium chloride), as the nitrogen concentration is 25-fold lower. This ratio may contribute to the differences in measured isopentenol production, since no production in M9 media was observed (Additional file 2A). These results illustrated that in terms of the C:N ratio, the observed isopentenol titer was limited to a defined concentration range in which strains could produce detectable isopentenol titers.

### Blocking organic acid formation yields limited improvements to isopentenol titer

**Fig. 4:**
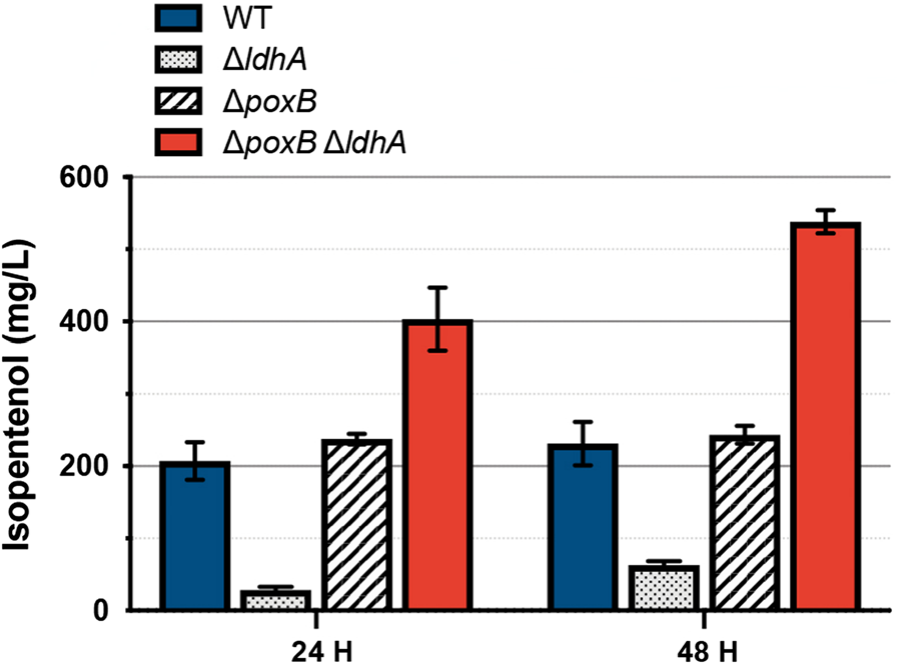
Rational engineering of *C. glutamicum* host chassis and isopentenol production pathway. Analysis of *C. glutamicum* strains ∆*poxB*, ∆*ldhA,* and double mutant ∆*poxB* ∆*ldhA* strains on isopentenol production: Strains of the indicated genotype (WT, ∆*poxB*, ∆*ldhA,* and ∆*poxB* ∆*ldhA*) were cultivated in 24-well deep well plates. Isopentenol titer was measured as described in Additional file 2A, B. Data was generated from three independent biological replicates for each genotype, and error bars indicate standard error

Acetate and lactate formation increased under the cultivation conditions we used for isopentenol production (Fig. 3a–c). To test if isopentenol titer could be improved by blocking formation of these organic acids, we targeted the genes encoding pyruvate oxidase (*poxB*) or lactate dehydrogenase (*ldhA*) for deletion. We generated the single mutant strains ∆*poxB* and ∆*ldhA,* as well as a double mutant strain ∆*poxB* ∆*ldhA* to redirect flux away from either or both acetate or lactate. Resulting strains were then transformed with the isopentenol production plasmid. The wild-type *C. glutamicum* produced ~ 225 mg/L at the 24 h timepoint, which was slightly lower than previous production runs with this strain but within the error range, likely due to batch to batch variation in media preparation (Fig. 4). The ∆*poxB* mutant did not significantly improve isopentenol titer (Fig. 4), even though accumulation of acetate was reduced from 5 g/L down to ~ 35 mg/L. The ∆*ldhA* strain did not produce more than trace isopentenol (Fig. 4). The ∆*poxB* ∆*ldhA* strain continued to show marked improvement over the wild-type strain at the 48 h timepoint, producing ~ 500 mg/L isopentenol in the ∆*poxB* ∆*ldhA* strain (Fig. 4). The rational gene deletions in *C. glutamicum* lowered accumulation of side-products, but improvements to isopentenol titer were limited to at most a twofold increase.

### Expression of the isopentenol pathway protein HmgR strongly correlates with isopentenol titer

To examine if isopentenol titers correlate with pathway protein expression, we used selected reaction monitoring (SRM) based proteomics [32–34] to assess relative protein levels across these conditions. Samples were grown in LB, M9, or CGXII media and collected at 24 and 48 h timepoints for proteomic analysis and GC analysis. We observed a similar dependence on the initial d-glucose concentration in CGXII media and a failure to produce isopentenol in M9 or LB irrespective of cultivation format (Additional file 4A). Crude lysates were prepared for peptide analysis to quantify the five proteins constituting the isopentenol pathway.

**Fig. 5:**
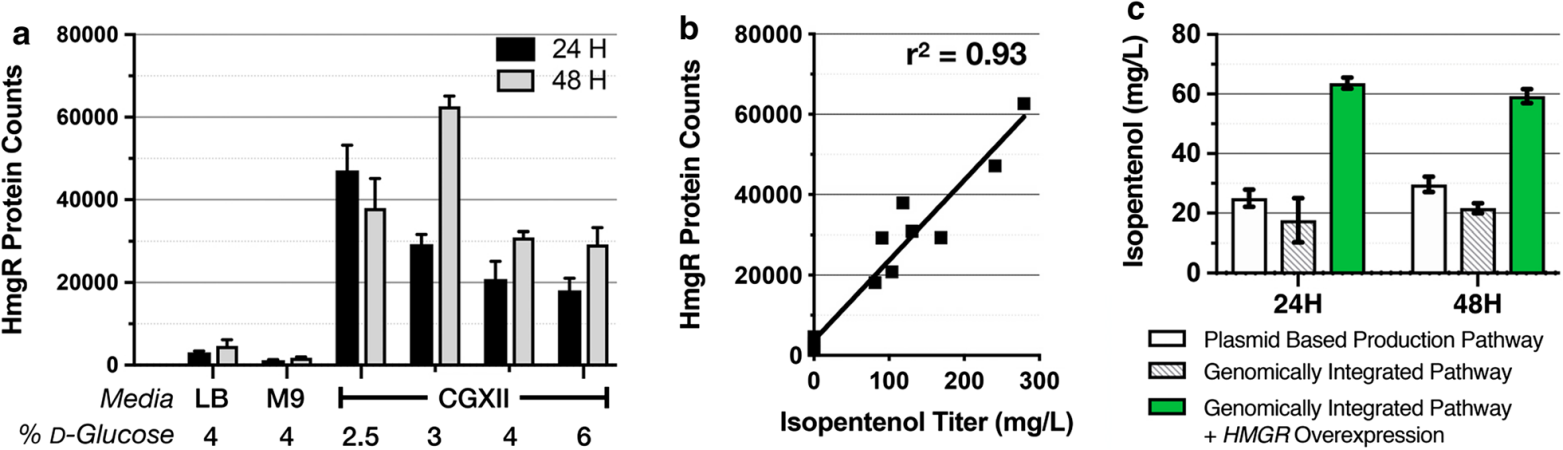
Analysis of HmgR Protein Abundance vs. Isopentenol Titer. All strains were prepared for isopentenol production in CGXII medium at the % (w/v) d-glucose concentration indicated below the graph. **a** Proteomic analysis of HmgR protein abundance: Absolute abundance for HmgR at the timepoints indicated are plotted as a function of cultivation condition. **b** Correlation analysis between isopentenol titer and HmgR protein abundance. Correlation was determined using a linear regression for the Pearson correlation coefficient (PCC) for the two variables. **c** Role of increased *HMGR* expression on isopentenol titer. All strains were cultivated for isopentenol production in CGXII medium amended with 4% d-glucose concentration in 24-well deep well plates. White bars: *C. glutamicum* ∆*idsA* harboring the plasmid-borne isopentenol production pathway. Grey hatched bars: ∆*idsA* with a chromosomally integrated isopentenol production pathway at the *idsA* locus. Green bars: ∆*idsA* with the same chromosomally encoded isopentenol production pathway as well as a plasmid-borne *HMGR* overexpression cassette. For all experiments, data was generated from three independent biological replicates for each condition, and error bars indicate standard error

The targeted protein analysis indicated the highest protein abundance in the optimal condition (CGXII with 3% d-glucose), whereas in LB, all proteins in the isopentenol production pathway were reduced in their abundance ranging from twofold (PMD) to tenfold (HmgR) lower (Fig. 5a and Additional file 4B). Pathway proteins from *C. glutamicum* grown in M9 media were reduced even further, as protein enrichment was decreased from sevenfold (PMD) to 27-fold (HmgS) (Fig. 5a and Additional file 4B). This analysis suggests that the failure to produce isopentenol in either LB or M9 media was due to the decreased abundance of the isopentenol pathway proteins.

Proteomics data also revealed potential bottlenecks in the isopentenol production pathway. Across the range of cultivation parameters and timepoints tested in wild-type *C. glutamicum*, we observed that HmgR protein levels and its isopentenol production titer were strongly correlated (r^2^ = 0.93) (Fig. 5b). The other four pathway proteins (AtoB, HmgS, MK, and PMD) were weakly correlated with the isopentenol titer (Additional file 4C).

To test if higher expression levels of *HMGR* could improve isopentenol production, we built a set of strains in which the isopentenol production pathway was chromosomally integrated at the *idsA* locus. The ∆*idsA* strains form white colonies instead of the wild-type yellow color [35] facilitating screening for heterologous pathway integration. The control ∆*idsA* strain harboring a plasmid-borne isopentenol production pathway produces ~ 20 mg/L isopentenol (Fig. 5c**)**. The ∆*idsA* strain with a chromosomally integrated isopentenol pathway was able to produce isopentenol, but less than the control strain with the plasmid-borne isopentenol pathway, at titers of ~ 12 mg/L (Fig. 5c). However, when HmgR expression was augmented in the chromosomally integrated production strain with an additional, plasmid-borne copy of *HMGR* under a *trc* promoter, the isopentenol titer improved 4× over the original strain and produced 60 mg/L isopentenol. While the cause of lower isopentenol production in the ∆*idsA* strains remain unclear, these results provide evidence consistent with the hypothesis where HmgR is a rate-limiting step in isopentenol production and provided a target for further pathway optimization.

### Production of isopentenol in *C. glutamicum* using IL-pretreated sorghum

**Fig. 6:**
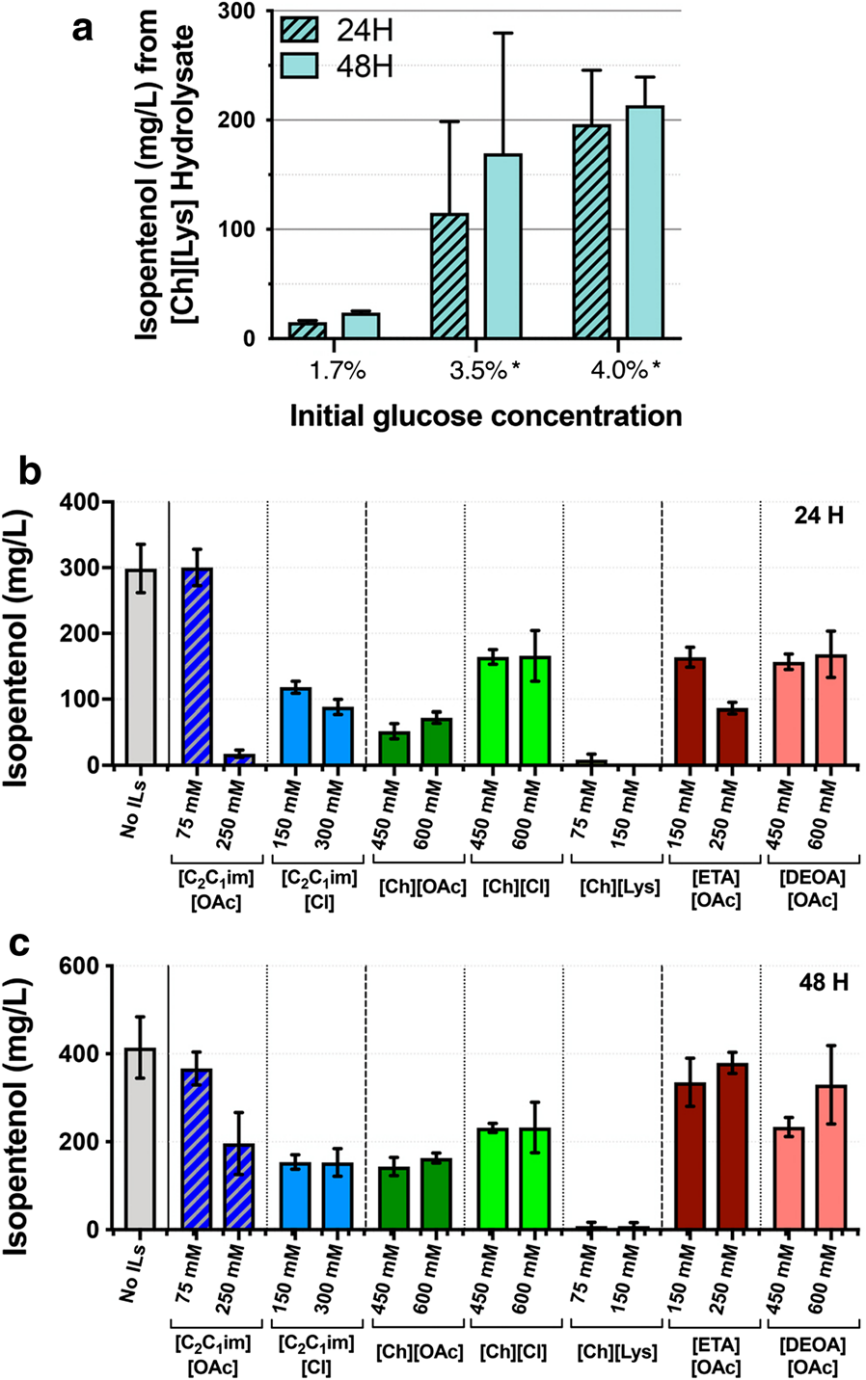
Isopentenol Production from [Ch][Lys] Pretreated Sorghum Hydrolysates as the Carbon Source. **a** *C. glutamicum* was prepared for isopentenol production in CGXII media with hydrolysate in 5 mL culture tubes. Additional d-glucose was supplemented as indicated (*). Isopentenol production values are the average of three independent biological replicates, and the error bars represent standard error. **b** Isopentenol production in the presence of ILs: CGXII minimal media including 4% starting d-glucose was used with or without IL. The IL concentrations tested are as listed on the *x* axis. Samples were cultivated in 24-well deep well plates. Data shown are after 24 h and 48 h of induction; the isopentenol production values are the average of biological triplicates, and the error bars represent standard error

To test this process with industrially relevant carbon sources, we demonstrated that *C. glutamicum* could produce isopentenol from plant biomass hydrolysate. We pretreated sorghum biomass using the IL [Ch][Lys] to generate a hydrolysate that contained 29.2 g/L d-glucose, 16.4 g/L d-xylose, and 5.1 g/L acetic acid (Additional file 5A, B). This hydrolysate also contained 0.01 mM 4-hydroxybenzoic acid and 1.36 mM benzoic acid (Additional file 5A, B). When the production strains were grown in the hydrolysate-amended CGXII media, no significant growth defect was observed compared to the growth in pure d-glucose supplemented CGXII media. Substituting hydrolysate for all added water in CGXII media results in a starting concentration of d-glucose at 1.7% (w/v). Based on our observations in Fig. 3a, we also prepared two other production conditions, where we supplemented pure d-glucose such that the concentration was increased to 3.5% or 4.0% (w/v). In the original hydrolysate-amended condition (with 1.7% d-glucose), we detected trace isopentenol production of ~ 15 mg/L (Fig. 6a). When the d-glucose concentration was increased to 3.5% or 4.0%, the titer increased to ~ 200 mg/L (Fig. 6a). Any potential contaminants from IL treated sorghum biomass also did not completely inhibit isopentenol production at these concentrations. These results demonstrate that *C. glutamicum* has the capacity to function as a microbial chassis for the production of terpenes from renewable starting materials.

### Ionic liquids do not inhibit isopentenol production

*Corynebacterium glutamicum* isopentenol production strains were tested for isopentenol production with tolerated levels of ILs (refer to Fig. 2a–c) higher than the 0.03% (w/v) remaining ILs after the ethanol/water washing regime [36]. Specifically, IL concentrations ranged from 75 mM to 600 mM, depending on where a growth defect was observed (Fig. 2a–c). After 24 h post-induction, all strains except for [Ch][Lys] reached similar high OD_600nm_ measurements; the [Ch][Lys] treated strains grew very poorly and did not reach saturation. Isopentenol production was measured both 24 and 48 h post-induction.

For [C_2_C_1_im][OAc], 75 mM had no impact on isopentenol production (Fig. 6b, dark blue bars). 250 mM [C_2_C_1_im][OAc] delayed isopentenol production, as no final product was recovered at 24 h, but the isopentenol titer was partially recovered at 48 h timepoint. Samples grown in the presence of [C_2_C_1_im][Cl] produced half as much isopentenol as the untreated control (Fig. 6b, light blue bars). As [Ch][Lys] was inhibitory to *C. glutamicum* growth (Fig. 2b), the strain failed to produce isopentenol in its presence (Fig. 6b, lime green bars). [Ch][OAc] and [Ch][Cl] had stronger effects on final product titer, decreasing isopentenol titer approximately 2–3× compared to the control, with [Ch][OAc] having a larger impact on final product titer than [Ch][Lys] (Fig. 6b, green bars). The reduction of isopentenol titer of these strains largely correlated with the severity of the growth defect, with the exception of [Ch][Cl], which exhibited no impact on doubling time but had a measurable impact on isopentenol production (refer to Fig. 2b).

In contrast to the cholinium and imidazolium based ILs, one of the protic ILs had modest effects on the production of isopentenol. Exogenous [DEOA][OAc] had a minor impact on isopentenol production (Fig. 6b, salmon pink bars), which is reasonably similar to [Ch][Cl]. However, strains grown in the presence of exogenous ethanolamine ([ETA][OAc]) had a similarly reduced titer at the 24 h timepoint, but the isopentenol titer was similar to production in the control strain when sampled at the 48 h timepoint (Fig. 6b, dark red bars). Taken together, these results indicate that *C. glutamicum* is competent to maintain the isopentenol titers in the presence of ILs and across several commonly used IL formats.

### Both strain background engineering and pathway engineering improve isopentenol titers

Additional *HMGR* expression improves isopentenol production (Fig. 5c). HmgR from *S. cerevisiae* is a class I HmG-CoA reductase, and preferentially uses NADPH as the cofactor rather than NADH [37, 38]. The use of a class II HmG-CoA reductase from *Silicibacter pomeroyi* which relies on NADH as a cofactor instead of NADPH has been reported previously [39]. We cloned the *S. pomeroyi hmgr* homolog into the isopentenol production plasmid and assessed isopentenol production in the ∆*poxB* ∆*ldhA* background. With the original isopentenol production pathway, *C. glutamicum* produces ~ 125 mg/L isopentenol with similar titers at both 24 h and 48 h timepoints (see Additional file 4A). In contrast, ∆*poxB* ∆*ldhA* strains showed a delay in initial isopentenol production, with lower production after 24 h (~ 100 mg/L) but an improvement over wild type to ~ 500 mg/L after 48 h (refer to Fig. 4).

**Fig. 7:**
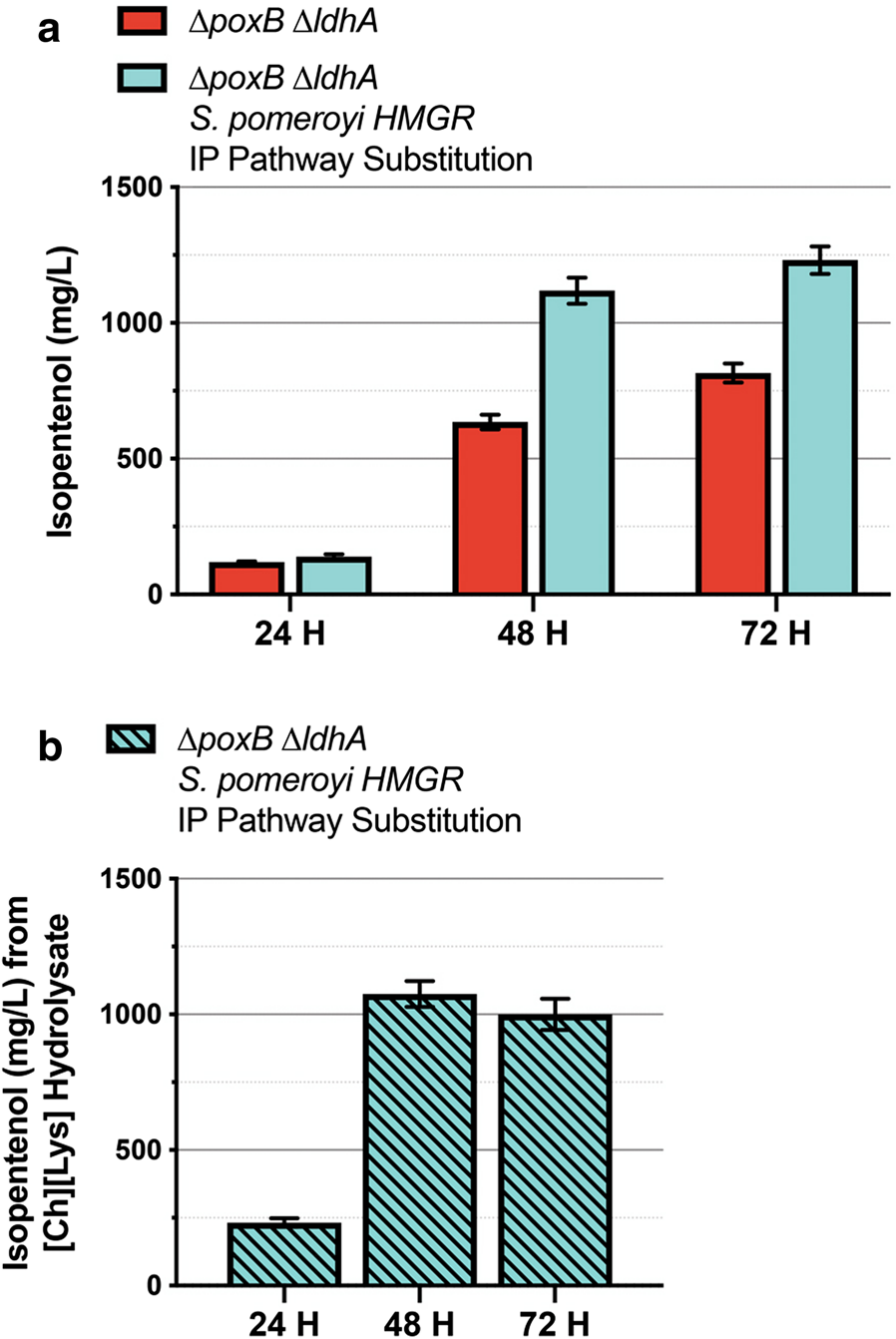
An Engineered *C. glutamicum* Host Chassis With Modified Isopentenol Production Pathway Produces Isopentenol Using IL-Pretreated Sorghum Biomass. **a** Analysis of the engineered isopentenol production pathway using a *hmgr* homolog from *S. pomeroyi* in a 5 mL cultivation format: Isopentenol production was performed in a *∆poxB* ∆*ldhA* strain harboring either the original isopentenol production pathway or a variant where the *S. cerevisiae HMGR* was replaced with a *hmgr homolog* from *S. pomeroyi*. One additional timepoint at 72 h post-induction was harvested. **b** Isopentenol production using IL-pretreated sorghum hydrolysate: CGXII minimal media was prepared as described previously in Fig. 5a. *C. glutamicum ∆poxB* ∆*ldhA* was adapted to growth in CGXII minimal media containing hydrolysate and assessed for isopentenol production at the timepoints indicated. All isopentenol values are the average of biological triplicates, and the error bars represent standard error

While titers were comparable at the 24 h timepoint with either the *S. cerevisiae HMGR* or *S. pomeroyi hmgr* genes by the 48 h timepoint, the *S. pomeroyi* variant strains produced close to 1120 mg/L of isopentenol (Fig. 7a). At the 72 h timepoint, we detected an increase to 1250 mg/L of isopentenol relative to ~ 750 mg/L from the unmodified heterologous pathway. This improvement in isopentenol production using the pathway variant suggests that the NADH-dependent allele of HmgR was advantageous for isopentenol production in *C. glutamicum*.

Finally, to complete our process characterization, we assayed isopentenol production from [Ch][Lys] pretreated hydrolysate with the optimized *C. glutamicum* ∆*poxB* ∆*ldhA* strain harboring the NADH-dependent *S. pomeroyi hmgr*, in the 5 mL cultivation format. The starting d-glucose concentration was supplemented to 4% as determined to be in the range for optimal final product titer. At the 48 h timepoint, the isopentenol production titer reached ~ 1100 mg/L, equivalent to the observed titer from when pure d-glucose was used as the carbon source (Fig. 7b). Our  % theoretical yield of isopentenol [40] from glucose was 9.7%, and  % theoretical yield from sorghum hydrolysate was 8.6%. These results demonstrate the completion of our microbial bioprocess for isopentenol production in the industrially relevant organism *C. glutamicum.*

## Discussion

Different microbial hosts have innate physiological differences that make them more or less suitable for a final bioconversion process. However, it is unclear a priori what parameters dictate final product titer of a given heterologous gene pathway. Here we have evaluated the advantages of an industrially relevant microorganism, *C. glutamicum,* to express a non-native mevalonate pathway consisting of five-genes with the goal of producing isopentenol from IL-pretreated plant biomass. Our highest isopentenol titers with sugars derived from plant biomass are comparable to titers published in *E. coli* which was cultivated in rich media with pure sugars [28, 41]. We also observed that *C. glutamicum* was natively resistant to a wide range of toxic compounds associated with the pretreatment process (i.e., ILs) and final product (i.e., isopentenol).

Carbon and nitrogen metabolism are closely linked through energy-driven processes such as the generation of ATP. Thus, the carbon to nitrogen ratio (C:N) is a critical parameter and its effect on growth and production has been reported in variety of microbial hosts [42, 43]. This study demonstrates a relationship between initial d-glucose concentrations in CGXII media and isopentenol production, which corresponds to a C:N ratio of 2.8–4.3 where we observed isopentenol production. A link between starting d-glucose concentration and its impact on gene expression (in the context of a heterologous gene pathway) has not been described before. This compositional profile indicates that common peptone-based media such as LB (or alternatively, defined M9 media) were not optimal for our purposes. Our observations are supported by evidence in *E. coli,* where cells exhibit differential transcriptional RNA levels and enzyme activities under nitrogen as well as carbon limitation [44]. However, it is still unclear how the initial cell physiology during exponential phase can impact the physiology after d-glucose exhaustion, where much of our batch-mode production occurs. A previous study also examined the impact of C:N ratio on the production of a native molecule [45], but testing a range of C:N values is not yet common practice when characterizing new products. The absolute starting d-glucose concentration (or C:N ratio) may be a general determinant of final product titer and could be beneficial for optimizing other heterologous gene pathways in *C. glutamicum*, such as for the production of the anthocyanin food colorant, cyanidin 3-*O*-glucoside [46].

Strain background engineering also played an important role in improving isopentenol titer. Having observed the undesirable formation of acetate and lactic acid in our isopentenol production experiments, we generated new strains to redirect metabolic flux away from these two organic acids. The single gene deletion strains ∆*ldhA* and ∆*poxB* did not accumulate their appropriate side-products, but also did not appreciably increase isopentenol titer. On the other hand, the isopentenol titer was increased in the double mutant (∆*poxB* ∆*ldhA*), which was a synergistic improvement over either of the single mutants. Genome-scale metabolic flux modeling and analysis [47] of these strains could reveal unappreciated nuances in host physiology which could inform future metabolic engineering work.

Finally, guided by proteomic analysis, pathway engineering resulted in the highest improvements in product titers to the grams per liter level. A strong correlation between the abundance of HmgR and isopentenol titer suggested HmgR as a potential rate-limiting step in this pathway. While increasing HmgR protein levels improved isopentenol production fourfold, the greatest absolute improvement of isopentenol titer was obtained by utilizing a class II NADH-dependent variant of HmgR from *S. pomeroyi* [39]. We suggest that additional mutagenesis of *HMGR* could lead to broadly applicable improvements for other molecules derived from the mevalonate pathway.

The system developed in this study sets the stage for characterization of this platform under simulated industrial bioreactor conditions, such as the scale down approach for 1,5‐diaminopentane [5]. In addition, technoeconomic analyses [48] are needed to fully understand the complex variables necessary to implement isopentenol production at an industrial scale. Currently, ILs are valuable and recycled after generating hydrolysates by extensive washing, and thus their concentration in hydrolysate is low [36, 49]. However, removal of ILs is not cost-effective in large-scale industrial applications. As they become less expensive, or used in consolidated one-pot processes [50, 51], host strains that can tolerate higher IL concentrations have the potential for an outsized impact on process cost. A higher d-glucose concentration in the hydrolysate could simplify the cultivation process we utilized for producing isopentenol from sorghum biomass hydrolysate.

## Conclusions

This report describes the successful deployment of a heterologous mevalonate-based pathway in the Gram-positive industrial microorganism, *C. glutamicum,* for the production of the biogasoline candidate and commodity chemical, isopentenol. Successful production of this final product was achieved at greater than 1 g/L from both pure glucose as well as IL-pretreated sorghum hydrolysate. We highlight the intrinsic capability of *C. glutamicum* as a valuable industrial host through characterizing phenotypic responses to emerging IL-based pretreatment reagents, and implementing genetic engineering and process optimization for terpene production.

## Materials and methods

### Chemicals and reagents

All chemicals and reagents were purchased from Sigma-Aldrich (St. Louis, MO) or as otherwise indicated, and were of molecular biology grade or higher. When cells were cultivated in a microtiter dish format, plates were sealed with a gas-permeable film (Sigma-Aldrich, St. Louis, MO).

### Strain and plasmid construction

All strains and plasmids used in this study are listed in Additional file 6: Table S1 and their sequences are available at http://public-registry.jbei.org. Oligo-nucleotide primers were synthesized by Integrated DNA Technologies, Inc. (San Diego, CA). Core primers used to validate genomic deletions are listed in Additional file 7: Table S2. Q5 High-Fidelity DNA Polymerase (New England Biolabs, Ipswich, MA) was used for polymerase chain reaction. Isothermal DNA assembly [52] was utilized to assemble plasmids using 40 nucleotide overhangs (NEBuilder HiFi DNA Assembly Master Mix, New England Biolabs, Ipswich, MA). Plasmids were constructed using chemically competent *E. coli* DH10β (New England Biolabs). Where indicated, the heterologous isopentenol production pathway was modified to incorporate the *hmgr* homolog from *Silicibacter pomeroyi* (NCBI: WP_011241944.1) to replace the existing gene from *S. cerevisiae* and the sequence was confirmed by Sanger sequencing. Integrated gene cassettes and gene deletions were confirmed by colony PCR to verify the complete excision at the targeted open reading frame using and inspected by agarose gel electrophoresis.

### Sorghum biomass pretreatment and regeneration

Whole commercial-grade sorghum plants derived from *Sorghum bicolor* were grown, harvested, and milled in the 2017 harvest cycle by Chromatin Inc (New Deal, Texas). This biomass was pretreated with [Ch][Lys] and afterwards, enzymatically saccharified for a total time of 72 h as described elsewhere [53]. To make CGXII amended with hydrolysate, CGXII was prepared using stock solutions of the individual components as described below. The hydrolysate was defrosted from storage at − 80 °C, filter sterilized through a 0.45-micron filter, and added in place of water in CGXII media. Detailed methods for the quantification of sugar and organic acid content is included in Additional file 8 (Additional Methods). Crystalline d-glucose was added to CGXII media with hydrolysate to increase the concentration up to 3.5% or 4% (w/v) as indicated.

### Preparation of electrocompetent *C. glutamicum* cells

*Corynebacterium glutamicum* was made electrocompetent as previously described [54]. In brief, cells were grown in NCM medium supplemented with 3% (v/v) glycine and electroporated with a Micro Pulser Electroporator (Bio-Rad Laboratories, Inc., Hercules, CA) at 10 μF, 600 Ω, and 1800 V. After electroporation cells were immediately mixed with 400 μL of BHIS broth and heat-shocked for 6 min at 46 °C. After a 2-h outgrowth at 30 °C, cells were plated on the appropriate selective media.

### Growth media composition

Isopentenol production was analyzed in several different common growth media. Lysogeny broth (LB): 10 g/L tryptone, 5 g/L yeast extract, and 5 g/L NaCl. Tryptone and yeast extract were purchased from BD Biosciences (Franklin Lakes, NJ). M9 minimal medium (Sambrook and Russell, 2001): 2 g/L (NH_4_)_2_PO_4_, 2 g/L KH_2_PO_4_, 1 g/L K_2_HPO_4_, 1 g/L NH_4_Cl, 0.5 g/L NaCl, 0.06 g/L MgSO_4_, 1.1 g/L CaCl_2_, 20 mg/L thiamine, and 0.2 mg/L biotin. CGXII minimal medium [30, 55]: 20 g/L (NH_4_)_2_SO_4_, 5 g/L urea, 1 g/L KH_2_PO_4_, 1 g/L K_2_HPO_4_, 0.25 g/L MgSO_4_·7H_2_O, 10 mg/L CaCl_2_, 10 mg/L FeSO_4_·7H_2_O, 10 mg/L MnSO_4_·H_2_O, 1 mg/L ZnSO_4_·7H_2_O, 0.2 mg/L CuSO_4_·5H_2_O, 0.02 mg/L NiCl_2_·6H_2_O, 0.2 mg/L biotin, 30 mg/L 3,4-dihydroxybenzoic acid, and 21 g/L 3-morpholinopropanesulfonic acid (MOPS); pH 7.0). d-Glucose was used as a carbon source at the % (w/v) concentration as indicated.

### Toxicity assays

To assess the impact of ILs on the growth of *C. glutamicum,* cells were first adapted to growth in CGXII media with 4% (w/v) d-glucose (described below). Then, cells were back-diluted into fresh CGXII media at a starting OD_600_ of 0.1 supplemented with the following ILs: 1-ethyl-3-methylimidazolium acetate ([C_2_C_1_im][OAc], also referred to as [EMIM][OAc]), 1-ethyl-3-methylimidazolium chloride ([C_2_C_1_im][Cl] also referred to as [EMIM][Cl]), cholinium acetate ([Ch][OAc]), cholinium chloride ([Ch][Cl]), cholinium lysinate ([Ch][Lys]), ethanol amine acetate [ETA][OAc], and diethanol amine acetate [DEOA][OAc] at the concentrations indicated. These cultures (100 µL volume per well) were grown at 30 °C in 96 well microtiter plates on a Synergy 4 plate reader (BioTek Instruments, Winooski VT) and shaken on the “high” setting. Optical density was tracked at a wavelength of 600 nm.

For the isopentenol toxicity assay, single colonies of *C. glutamicum* and *E. coli* were inoculated in 5 mL LB and grown overnight at 30 °C with shaking at 200 rpm. Each strain was serially diluted, inoculated on LB agar plates containing 2% (w/v) isopentenol. Photomicrographs were taken after 2 days of incubation at 30 °C.

### Isopentenol consumption or evaporation

Isopentenol evaporation was quantified in a 5 mL culture tube format and 24-well deep well plate format as described in [15]. To measure isopentenol consumption by *C. glutamicum*, wild-type *C. glutamicum* precultures were prepared in LB and back-diluted to an initial OD_600_ of 0.1 into fresh LB spiked with a serial dilution of the commercial standard isopentenol. Remaining isopentenol was quantified as described in the following section.

### Cultivation of *C. glutamicum* for isopentenol production

All cells taken from − 80 °C glycerol stocks were plated on LB agar plates containing the appropriate antibiotic. A single colony was inoculated and grown overnight in 5 mL LB at 30 °C for *C. glutamicum* on a rotary shaker at 200 rpm. Where necessary, kanamycin was added to growth media at a final concentration of 50 μg/mL. Unless otherwise noted, all seed cultures were first inoculated for growth in culture tubes. If cells were grown in a 24-well deep well format, 2 mL of culture media was used per well. Deep well plates were incubated Infors Multitron Incubator with a 3 mm Orbital Shaking Platform shaken at 999 rpm (Bottmingen, Switzerland). When grown in a 5 mL culture tube format, 5 mL of culture media was used per cultivation. Cultures were shaken at 200 rpm on a platform shaker. All strains were cultivated under aerobic growth conditions.

When grown in rich media, the heterologous isopentenol production pathway was induced when the OD_600_ reached ~ 0.8 with 500 µM IPTG without adaptation. When minimal media was used, cells from a seed culture were sub-cultured twice to adapt cells to growth in the media. In brief, pre-cultured cells in LB were diluted 1:10 into minimal media and grown overnight at 30 °C with shaking at 200 rpm using deep well plates. The adapted cultures were then diluted 1:10 into fresh minimal media and grown overnight at 30 °C with the same cultivation format. After a second back dilution to allow for complete adaptation to growth in minimal media, cultures were then used for growth assays and production in minimal media. The d-glucose concentration was held constant throughout all passaging steps required for adaptation. Where hydrolysate was used as the carbon source, CGXII media was prepared, substituting the volume used for dH_2_O with the hydrolysate. Crystalline d-glucose was added to this solution to increase the starting d-glucose concentration. The isopentenol production pathway was induced at the same OD_600_ as with rich media, and with the same concentration of IPTG at 500 µM unless otherwise indicated.

To assess isopentenol production under IL stress conditions, the adapted cultures of *C. glutamicum* were first back-diluted to OD_600_ of 0.1 into CGXII minimal medium including 4% (w/v) d-glucose and the ILs at the concentrations described above. The production pathway was then induced as before when cultures reached an OD_600_ of ~ 0.8.

### Analytical methods for isopentenol quantification

To quantify isopentenol, 300 μL of cell culture was added to 300 μL of ethyl acetate containing n-butanol (10 μg/L) as an internal standard and processed as described previously [28]. Briefly, sample mixtures were shaken at maximum speed for 15 min using an MT-400 microtube mixer (TOMY Seiko, Tokyo, Japan) and then centrifuged at 14,000*g* for 3 min to separate the organic phase from the aqueous phase. 60 μL of the organic layer was transferred into an Agilent glass insert placed inside of a GC vial and 1 μL was analyzed by Agilent GCMS equipped with a DB-5 column (Agilent Technologies, Santa Clara, CA) or Thermo GC-FID equipped with a DB-WAX column (Agilent Technologies) for quantification of isopentenol. Analytical grade standards were used to calculate analyte concentrations and confirm identification of peaks. Sugars and organic acids were quantified exactly as described in [56].

Reported isopentenol titers from experiments conducted in the 24-well deep well plate were corrected for evaporation at the 48 h timepoint by accounting for the amount of product lost from the 24 h timepoint. The reported isopentenol titers for the 48 h timepoint are the sum of the amount detected by GC analysis plus the estimated isopentenol lost from the 24 h timepoint. Product loss from evaporation was estimated using a fitted logarithmic curve from the evaporation rates determined in Additional file 1D with different isopentenol starting concentrations after 24 h incubation. The formula used was *y* = − 0.129ln(x) + 1.5375 and *r*^2^ = 0.9833.

### Proteomics

A targeted SRM (selected reaction monitoring) method was developed to quantify relative levels of pathway proteins in samples under the various tested conditions in a 5 mL cultivation format. At the timepoints indicated, 1 mL of each sample was pelleted by centrifugation at 14,000*g* and flash frozen with liquid nitrogen at − 80 °C until ready for processing. Cells were lysed in 100 mM NaHCO_3_ using 0.1 mm glass beads using a Biospec Beadbeater (Biospec Products, Bartlesville, OK) with 60 s bursts at maximum power and repeated three times. Cell lysates were cooled on ice between each round. The clarified supernatant was harvested by centrifugation at 14,000*g* and the soluble protein concentration was determined with the BCA method (ThermoFisher Scientific/Pierce Biotechnology, Waltham, MA). The SRM-targeted proteomic assays and analyses were performed as described previously [57], on an Agilent 6460 QQQ mass spectrometer system coupled with an Agilent 1290 UHPLC system (Agilent Technologies, Santa Clara, CA). Equal amount (20 μg) of peptides in each sample were loaded and separated on an Ascentis Express Peptide C18 column [2.7-mm particle size, 160-Å pore size, 5-cm length × 2.1-mm inside diameter (ID), coupled to a 5-mm × 2.1-mm ID guard column with the same particle and pore size, operating at 60 °C; Sigma-Aldrich] operating at a flow rate of 0.4 mL/min via the following gradient: initial conditions were 98% solvent A (0.1% formic acid), 2% solvent B (99.9% acetonitrile, 0.1% formic acid). Solvent B was increased to 35% over 6.5 min, then increased to 80% over 1.5 min, and held for 1.5 min at a flow rate of 0.6 mL/min, followed by a ramp back down to 2% of B over 0.5 min where it was held for 1 min to re-equilibrate the column to original conditions. The data were acquired using Agilent MassHunter version B.08.02. Acquired SRM data were analyzed by Skyline software version 3.70 (MacCoss Lab Software). The SRM methods and data are available at Panoramaweb [58] (https://goo.gl/GgQGns). Peptide abundances of the same protein were summed together to assign the protein abundance of a given protein. The average protein value is shown from samples in biological triplicate to assess different timepoints and growth conditions.

## Additional files

**Additional file 1.** Evaluation of Production Condition and *C. glutamicum* Properties as the Isopentenol Production Chassis. **(A-B)** Growth assay of *E. coli* and *C. glutamicum* to ILs; 0–300 mM [C_2_C_1_im][OAc] and 0–40 mM [Ch][Lys] in LB in 96 well microtiter plates. **(C)** Analysis of isopentenol evaporation or consumption by *C. glutamicum* in a 5 mL cultivation format. **(D)** The same evaporation assay as in (C), but in a 24 well format.

**Additional file 2.** Isopentenol Production in *C. glutamicum* Strains Cultivated in Rich vs. Minimal Media. **(A)** Isopentenol production in minimal media: *C. glutamicum* was prepared for isopentenol production in two minimal media; M9 and CGXII supplemented with either 1% or 4% d-glucose as the carbon source in 5 mL tubes. **(B)** Isopentenol production in rich media: *C. glutamicum* was prepared for isopentenol production in LB media supplemented with either 1% or 4% d-glucose as the carbon source in a 5 mL culture tube. Data shown are production 48 h after induction and is the average of biological triplicates; error bars represent standard error.

**Additional file 3.** Impact of Initial Glucose and Nitrogen Concentrations On Isopentenol Production in *C. glutamicum.* **(A)** Analysis of residual d-glucose in CGXII media with a range of starting d-glucose concentrations as indicated in 24-well deep well plates. **(B)** Analysis of the generated succinate titer during isopentenol production in Fig. 3A. **(C)** Correlation of OD_600_ of samples grown at different d-glucose concentrations with isopentenol titer at the 24 h timepoint. Correlation was determined using linear regression for the Pearson correlation coefficient (PCC) for the two variables, and r^2^ = 0.00053, and is indicated with a solid black line. **(D)** Impact of different nitrogen concentrations on isopentenol production: *C. glutamicum* was cultivated for isopentenol production in CGXII media, where the nitrogen concentration was varied from 20.3 mM to 1120 mM at the fixed d-glucose concentration of 220 mM. **(E)** Visualization of carbon:nitrogen (C:N) ratio: The C:N ratio ranged from 0.1 to 32.8. For simplicity, the potential contribution of carbon from 3,4-dihydroxybenzoic acid was excluded from this calculation. When cultivated with 5.5 mM d-glucose in CGXII media, *C. glutamicum* showed poor growth. No other gross differences in biomass were noted at other conditions.

**Additional file 4.** Analysis of Pathway Protein Abundance vs. Isopentenol Titer in Three Kinds of Media. **(A)** *Left*. Analysis of isopentenol titer measured in wild-type *C. glutamicum*. Production of isopentenol from *C. glutamicum* in CGXII media with d-glucose concentrations as indicated. *Right*. The same strain was cultivated for isopentenol production in LB with 4% d-glucose, or M9 media with 4% d-glucose. The production of isopentenol from CGXII media with 3% d-glucose is replotted from the left-hand graph for ease of comparison. The relevant media and % d-glucose are indicated below the graph. Data was generated from three independent biological replicates for each condition and the error bars indicate standard error. **(B)** Proteomic analysis of AtoB, HmgS, MK, and PMD protein abundances: Each protein abundance is shown at the 24 h and 48 h timepoint in wild-type *C. glutamicum* cultivated with 4% starting d-glucose in LB and M9 media, and 2.5–6% starting d-glucose in CGXII media. **(C)** Correlation between isopentenol titer and each protein abundance at the 24 h timepoint. Correlation was determined using linear regression for the Pearson correlation coefficient (PCC) for the two variables. Cultivations for proteomics samples were performed as described in “Materials and methods” section.

**Additional file 5.** Determination of sugars and aromatics in hydrolysate by HPLC. **(A)** *Left Panel.* Standards for aromatics (0.5 g/L of each compound): A = 4-hydroxybenzoic acid; B = vanillic acid; C = *p*-coumaric acid; D = ferulic acid; E = vanillin; F = benzoic acid. *Right Panel*. Organic acids standards (1 g/L of each compound): G = lactic acid; H = formic acid; I = acetic acid. **(B)** Representative traces for aromatics and organic acids from [Ch][Lys] pretreated hydrolysate. *Left Panel.* Aromatics. Peaks are numbered as follows as identified in hydrolysate: 1 = 4-hydroxybenzoic acid; 2 = benzoic acid. *Right Panel.* Sugars and organic acids identified in hydrolysate: 3 = d-glucose; 4 = d-xylose; 5 = lactic acid; 6 = acetic acid. Concentrations of the sugars and aromatics from the [Ch][Lys] pretreated sorghum biomass were as follows. d-Glucose: 29.2 g/L; d-xylose: 16.4 g/L; acetic acid: 5.1 g/L; lactic acid: 6.69 g/L; 4-hydroxybenzoic acid: 0.0018 g/L; benzoic acid: 0.167 g/L. The analytes for vanillic acid, *p*-coumaric acid, ferulic acid, and vanillin were detected but below the linear range for quantification (< 1 mg/L).

**Additional file 6.** Strains and plasmids used in this study.

**Additional file 7.** Genotyping primers.

**Additional file 8.** Additional Methods. Determination of sugars, organic acids and monomeric aromatics in the hydrolysate.

## Abbreviations

IL: ionic liquid
[C_2_C_1_im]: 1-ethyl-3-methylimidazolium
[Ch]: cholinium
[ETA][OAc]: ethanolamine acetate
[DEOA][OAc]: diethanolamine acetate
LB: lysogeny broth
SRM: selected reaction monitoring
