# Supplementary figures and images for: Engineering Corynebacterium glutamicum to produce the biogasoline isopentenol from plant biomass hydrolysates
Source: Biotechnol Biofuels. 2019 Feb 27;12:41. doi: 10.1186/s13068-019-1381-3 (PMC6391826; doi:10.1186/s13068-019-1381-3)

AF1A.

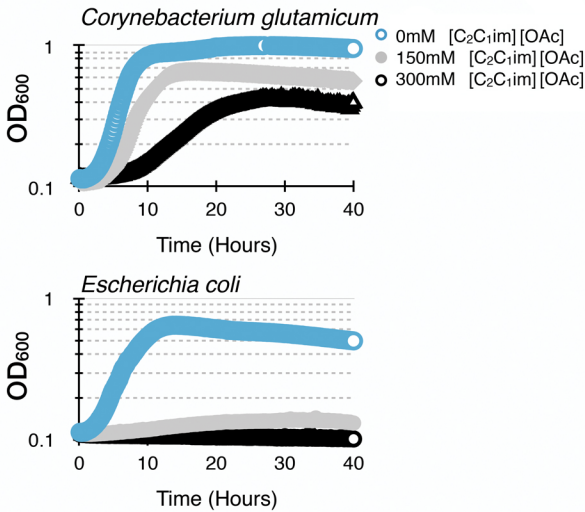

AF1B.

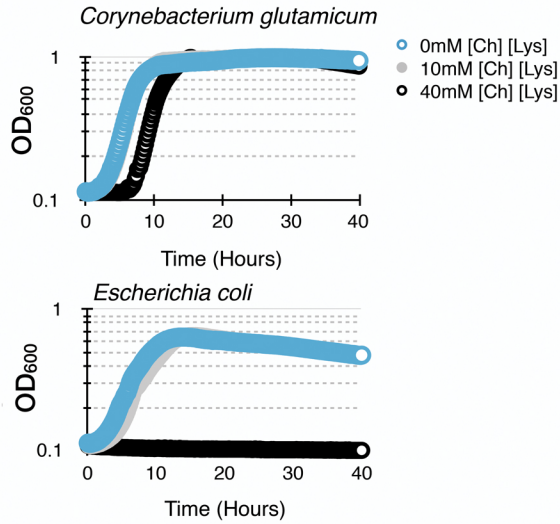

AF1C.

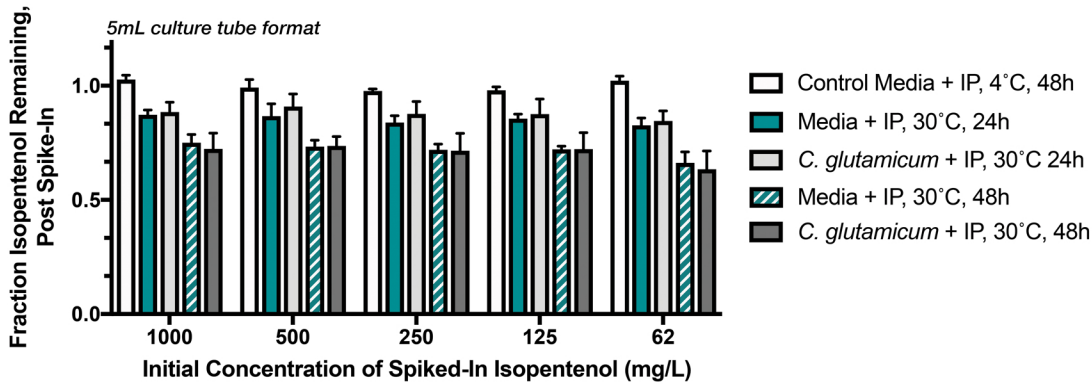

AF1D.

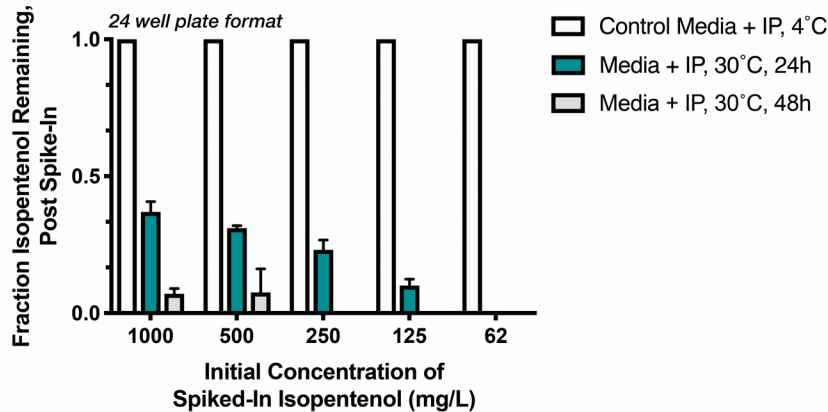

Supplement: Supplementary file 1 — Additional file 1. Evaluation of Production Condition and C. glutamicum Properties as the Isopentenol Production Chassis. (A-B) Growth assay of E. coli and C. glutamicum to ILs; 0–300 mM [C2C1im][OAc] and 0–40 mM [Ch][Lys] in LB in 96 well microtiter plates. (C) Analysis of isopentenol evaporation or consumption by C. glutamicum in a 5 mL cultivation format. (D) The same evaporation assay as in (C), but in a 24 well format. [file 13068_2019_1381_MOESM1_ESM.pdf]

# AF2A.

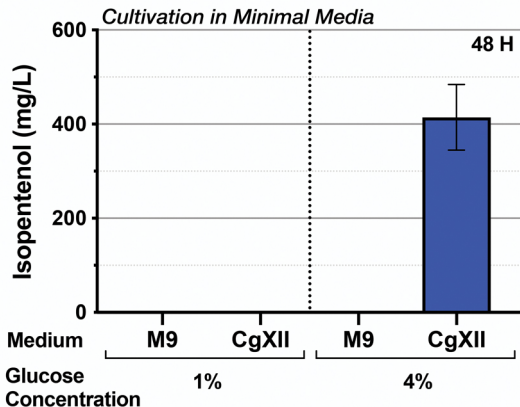

# AF2B

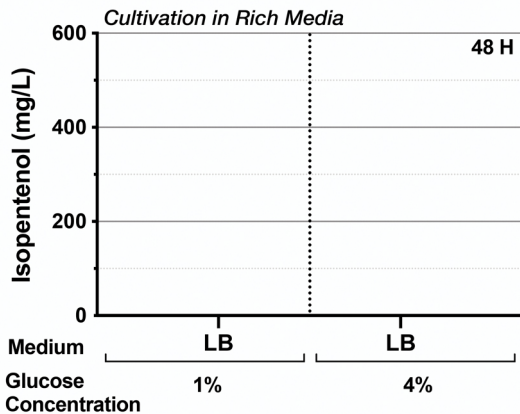

Supplement: Supplementary file 2 — Additional file 2. Isopentenol Production in C. glutamicum Strains Cultivated in Rich vs. Minimal Media. (A) Isopentenol production in minimal media: C. glutamicum was prepared for isopentenol production in two minimal media; M9 and CGXII supplemented with either 1% or 4% d-glucose as the carbon source in 5 mL tubes. (B) Isopentenol production in rich media: C. glutamicum was prepared for isopentenol production in LB media supplemented with either 1% or 4% d-glucose as the carbon source in a 5 mL culture tube. Data shown are production 48 h after induction and is the average of biological triplicates; error bars represent standard error. [file 13068_2019_1381_MOESM2_ESM.pdf]

**AF3A.**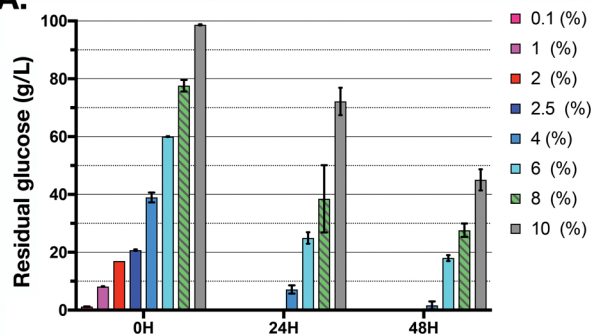**AF3B.**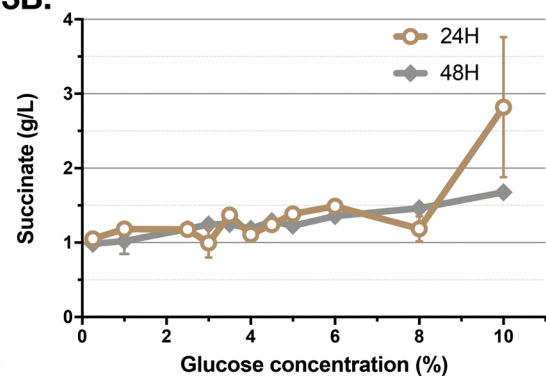**AF3C.**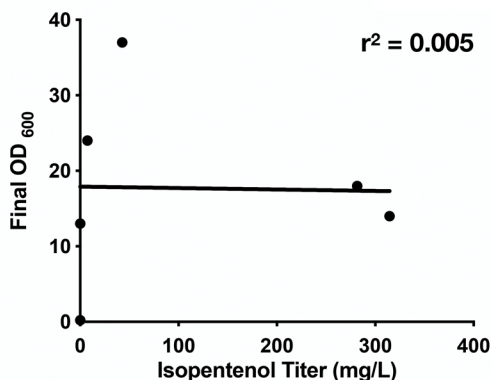**AF3D.**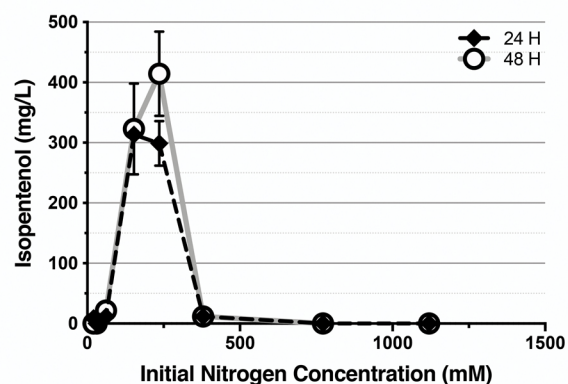**AF3E.**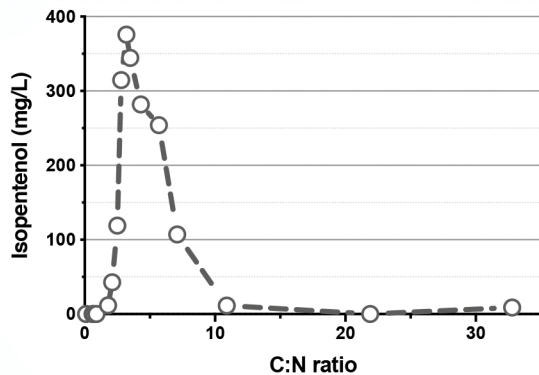

Supplement: Supplementary file 3 — Additional file 3. Impact of Initial Glucose and Nitrogen Concentrations On Isopentenol Production in C. glutamicum. (A) Analysis of residual d-glucose in CGXII media with a range of starting d-glucose concentrations as indicated in 24-well deep well plates. (B) Analysis of the generated succinate titer during isopentenol production in Fig. 3A. (C) Correlation of OD600 of samples grown at different d-glucose concentrations with isopentenol titer at the 24 h timepoint. Correlation was determined using linear regression for the Pearson correlation coefficient (PCC) for the two variables, and r2 = 0.00053, and is indicated with a solid black line. (D) Impact of different nitrogen concentrations on isopentenol production: C. glutamicum was cultivated for isopentenol production in CGXII media, where the nitrogen concentration was varied from 20.3 mM to 1120 mM at the fixed d-glucose concentration of 220 mM. (E) Visualization of carbon:nitrogen (C:N) ratio: The C:N ratio ranged from 0.1 to 32.8. For simplicity, the potential contribution of carbon from 3,4-dihydroxybenzoic acid was excluded from this calculation. When cultivated with 5.5 mM d-glucose in CGXII media, C. glutamicum showed poor growth. No other gross differences in biomass were noted at other conditions. [file 13068_2019_1381_MOESM3_ESM.pdf]

AF4A.

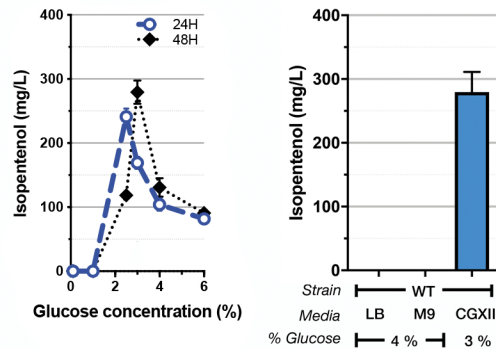

AF4B.

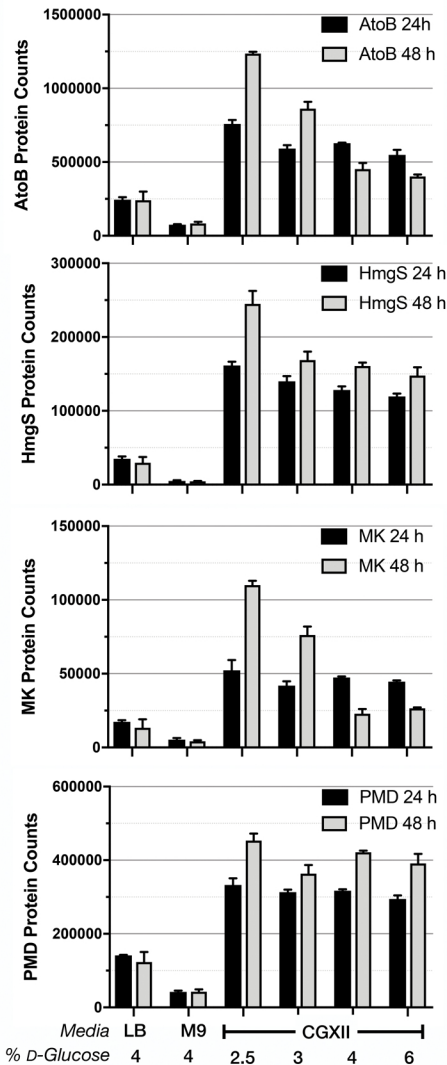

AF4C.

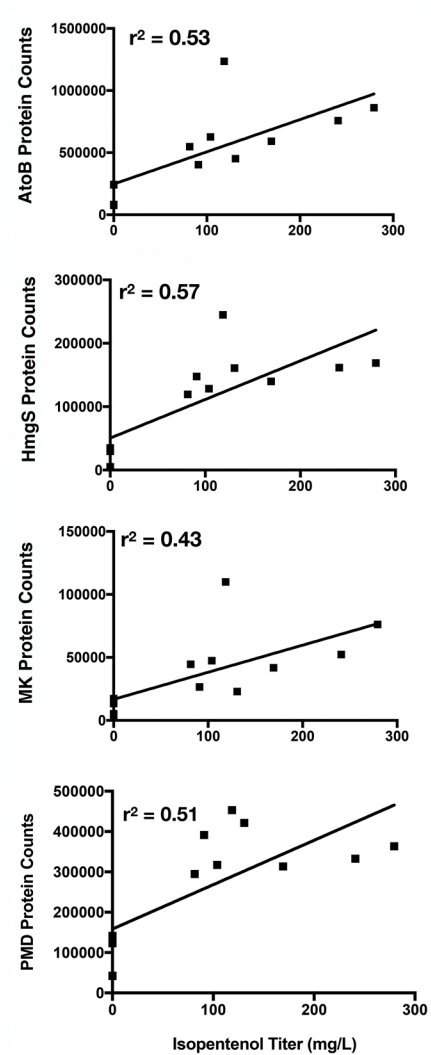

Supplement: Supplementary file 4 — Additional file 4. Analysis of Pathway Protein Abundance vs. Isopentenol Titer in Three Kinds of Media. (A) Left. Analysis of isopentenol titer measured in wild-type C. glutamicum. Production of isopentenol from C. glutamicum in CGXII media with d-glucose concentrations as indicated. Right. The same strain was cultivated for isopentenol production in LB with 4% d-glucose, or M9 media with 4% d-glucose. The production of isopentenol from CGXII media with 3% d-glucose is replotted from the left-hand graph for ease of comparison. The relevant media and % d-glucose are indicated below the graph. Data was generated from three independent biological replicates for each condition and the error bars indicate standard error. (B) Proteomic analysis of AtoB, HmgS, MK, and PMD protein abundances: Each protein abundance is shown at the 24 h and 48 h timepoint in wild-type C. glutamicum cultivated with 4% starting d-glucose in LB and M9 media, and 2.5–6% starting d-glucose in CGXII media. (C) Correlation between isopentenol titer and each protein abundance at the 24 h timepoint. Correlation was determined using linear regression for the Pearson correlation coefficient (PCC) for the two variables. Cultivations for proteomics samples were performed as described in “Materials and methods” section. [file 13068_2019_1381_MOESM4_ESM.pdf]

**AF5A.**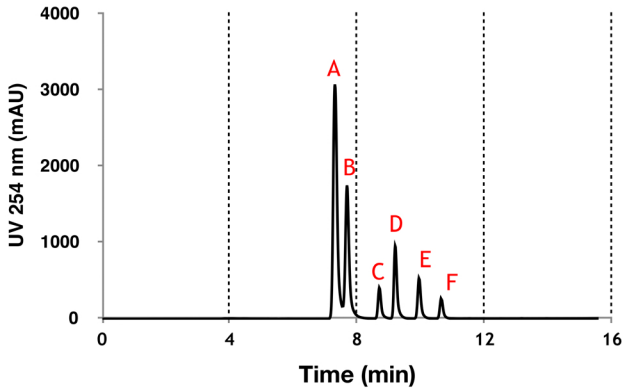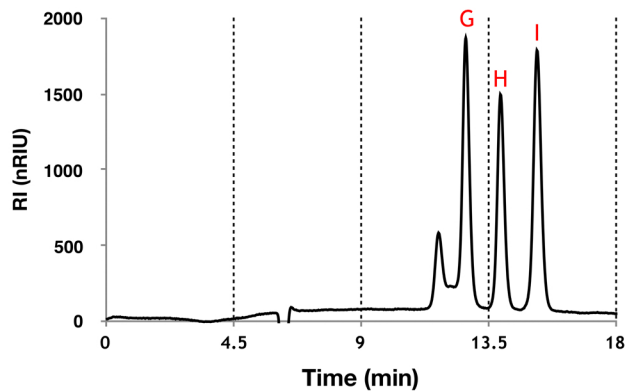**AF5B.**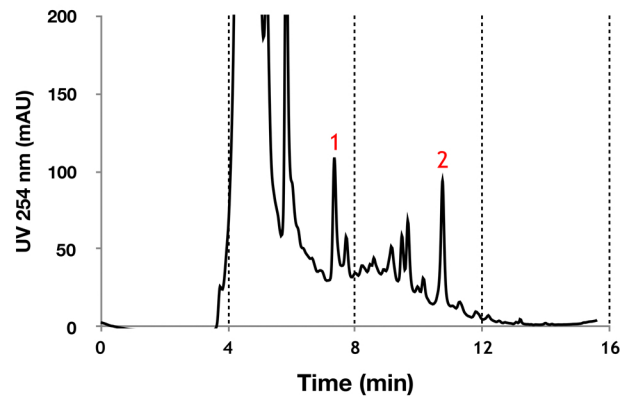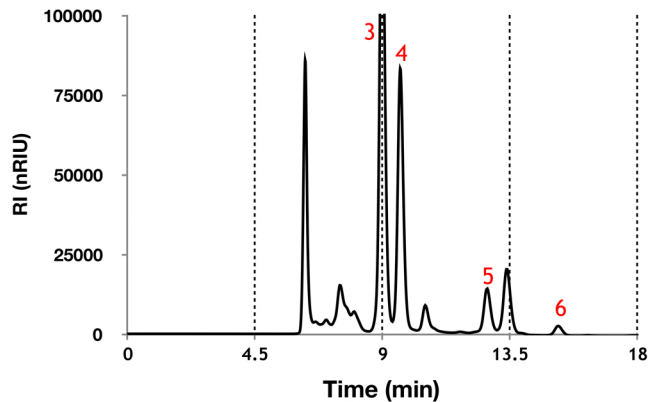

Supplement: Supplementary file 5 — Additional file 5. Determination of sugars and aromatics in hydrolysate by HPLC. (A) Left Panel. Standards for aromatics (0.5 g/L of each compound): A = 4-hydroxybenzoic acid; B = vanillic acid; C = p-coumaric acid; D = ferulic acid; E = vanillin; F = benzoic acid. Right Panel. Organic acids standards (1 g/L of each compound): G = lactic acid; H = formic acid; I = acetic acid. (B) Representative traces for aromatics and organic acids from [Ch][Lys] pretreated hydrolysate. Left Panel. Aromatics. Peaks are numbered as follows as identified in hydrolysate: 1 = 4-hydroxybenzoic acid; 2 = benzoic acid. Right Panel. Sugars and organic acids identified in hydrolysate: 3 = d-glucose; 4 = d-xylose; 5 = lactic acid; 6 = acetic acid. Concentrations of the sugars and aromatics from the [Ch][Lys] pretreated sorghum biomass were as follows. d-Glucose: 29.2 g/L; d-xylose: 16.4 g/L; acetic acid: 5.1 g/L; lactic acid: 6.69 g/L; 4-hydroxybenzoic acid: 0.0018 g/L; benzoic acid: 0.167 g/L. The analytes for vanillic acid, p-coumaric acid, ferulic acid, and vanillin were detected but below the linear range for quantification (< 1 mg/L). [file 13068_2019_1381_MOESM5_ESM.pdf]
