# Supplementary material for: Engineering Corynebacterium glutamicum to produce the biogasoline isopentenol from plant biomass hydrolysates
Source: Biotechnol Biofuels. 2019 Feb 27;12:41. doi: 10.1186/s13068-019-1381-3 (PMC6391826; doi:10.1186/s13068-019-1381-3)
Supplement: Supplementary file 6 — Additional file 6. Strains and plasmids used in this study. [file 13068_2019_1381_MOESM6_ESM.docx]

**Table S1** Strains and plasmids used in this study.

| **Strain** | **Description** | **Selection** | **Reference** |
| --- | --- | --- | --- |
| JBEI-7936 | *Corynebacterium glutamicum* ATCC 13032 / NHRI 1.1.2, biotin auxotroph | Nx^R^ | Gift from Jay Keasling |
| JBEI-19571 | JBEI-7936 harboring p/JBEI-19559 | Kan^R^ | This study |
| JBEI-19652 | JBEI-7936 harboring p/JBEI-19628 | Kan^R^ | This study |
| JBEI-19658 | JBEI-7936 harboring p/JBEI-19634 | Kan^R^ | This study |
| JBEI-19563 | JBEI-7936 Δ*poxB* | Suc^R^, Kan^S^ | This study |
| JBEI-19566 | JBEI-7936 Δ*poxB* Δ*ldhA* | Suc^R^, Kan^S^ | This study |
| JBEI-19572 | JBEI-19566 harboring p/JBEI-19559 | Kan^R^ | This study |
| JBEI-19646 | JBEI-19566 harboring p/JBEI-19625 | Kan^R^ | This study |
| JBEI-19655 | JBEI-7936 *cg1122:P_lacI_-lacI-P_lacUV5_–lacZα-T7 gene1:cg1121* “(DE3)” | Suc^R^, Kan^S^ | This study |
| JBEI-19654 | JBEI-19655 harboring p/JBEI-19632 | Kan^R^ | This study |
| JBEI-18084 | *C. glutamicum ∆cgIIM* ∆*cgIIR* ∆*cgIIIR* (methylation deficient strain) | Nx^R^ | This study |
| JBEI-19703 | JBEI-18084 Δ*idsA* | Suc^R^, Kan^S^ | This study |
| JBEI-19702 | JBEI-19703 p/JBEI-19559 | Kan^R^ | This study |
| JBEI-19657 | *C. glutamicum ∆cgIIM* ∆*cgIIR* ∆*cgIIIR* Δ*idsA::P_LacUV5_*-*atoB*-*ScHMGS*-*ScHMGR*-*P_trc_*-*MK*-*ScPMD* | Suc^R^, Kan^S^ | This study |
| JBEI-19656 | JBEI-19657 harboring p/JBEI-19626 (“HMGR augmented”) | Kan^R^ | This study |
| *E. coli DH1* | *F^–^ λ^–^ endA1 recA1 relA1 gyrA96 thi-1 glnV44 hsdR17(r_K_^–^m_K_^–^)* |  | Meselson and Yuan, 1968 |
| *E. coli DH10β* | F^–^ *endA1* *deoR*^+^ *recA1* *galE15* *galK16* *nupG* *rpsL* Δ*(lac)X74* φ80*lacZΔM15* *araD139* Δ*(ara,leu)7697* *mcrA* Δ*(mrr-hsdRMS-mcrBC)* Str^R^ λ^–^ |  | Invitrogen |
| **Plasmid** | **Description** | **Selection** | **Reference** |
| JBEI-2600 | pEC-XK99E, *E. coli*-*C. glutamicum* shuttle expression vectors based on the medium-copy number plasmid including *pGA1*, Kan^R^, *ori*V, P*_trc_* | Kan^R^ | Kirchner et al., 2003 |
| pK18mobsacB | *sacB* counter-selection plasmid, *ori*V origin of replication | Kan^R^ | Schafer et al., 1994 |
| JBEI-19558 | pK18mobsacB-∆*idsA* | Suc^s^, Kan^R^ | This study |
| JBEI-19556 | pK18mobsacB-∆*poxB* | Suc^s^, Kan^R^ | This study |
| JBEI-19557 | pK18mobsacB-∆*ldhA* | Suc^s^, Kan^R^ | This study |
| JBEI-9321 | pA5c-MevT(O)-T21-MKco-PMDsc “IPP-bypass pathway” | Cm^R^ | Kang et al., 2016 |
| JBEI-19559 | pEC-XK99E-AK-IP-bypass | Kan^R^ | This study |
| JBEI-19628 | pTE221 pEC-XK99E-AK-IP-bypass-*S. aureus mvaK1, mvaS* (substitution) | Kan^R^ | This study |
| JBEI-19634 | pTE222 pEC-XK99E-AK-IP-bypass-*C. kroppenstedtii mvaK1, mvaA* (substitution) | Kan^R^ | This study |
| JBEI-19625 | pTE202 pEC-XK99E-AK-IP-bypass-*S. pomeroyi* *HMGR* (substitution) | Kan^R^ | This study |
| JBEI-19632 | pTE155 pEC-XK99E-AK-IP-bypass, T7 promoter | Kan^R^ | This study |
| JBEI-19626 | pLacUV5-*HMGR* | Kan^R^ | This study |
|  |  |  |  |
|  |  |  |  |
|  |  |  |  |
|  |  |  |  |
|  |  |  |  |
|  |  |  |  |
