# Supplementary material for: Engineering Corynebacterium glutamicum to produce the biogasoline isopentenol from plant biomass hydrolysates
Source: Biotechnol Biofuels. 2019 Feb 27;12:41. doi: 10.1186/s13068-019-1381-3 (PMC6391826; doi:10.1186/s13068-019-1381-3)
Supplement: Supplementary file 7 — Additional file 7. Genotyping primers. [file 13068_2019_1381_MOESM7_ESM.docx]

**Additional Table S2** Primers used in this study

| **Primer Name** | **Sequence** | **Forward/Reverse:Plasmid name** |
| --- | --- | --- |
| TEAM-13 | ATCACGGATCCGCGTCATGAAGTGCAGATCTTCCGCACCTTCGG | F-insert1: JBEI-19558 |
| TEAM-14 | ATGCAGTTTAAACGGAGACGTCCTTCAATCGAATAGGGG | R-insert1: JBEI-19558 |
| TEAM-15 | GAGCCAATAATGCGCACTAGCTCTAGAGTCGACCTGCAGGC | F-insert2: JBEI-19558 |
| TEAM-16 | CCCGCGGCAGAAACAATTAGCCCGGGTACCGAGCT | R-insert2: JBEI-19558 |
| TEAM-1022 | GAGCCAATAATGCGCACTAGCTCTAGAGTCGACCTGCAGGC | F-vector: JBEI-19556 |
| TEAM-1023 | CCCGCGGCAGAAACAATTAGCCCGGGTACCGAGCT | R-vector: JBEI-19556 |
| TEAM-1024 | ATTCGAGCTCGGTACCCGGGCTAATTGTTTCTGCCGCGGG | F-insert1: JBEI-19556 |
| TEAM-1025 | CATAACGTTGAGGAGTTCAGGTGGGAACAGGTCATGGGATTCAG | R-insert1: JBEI-19556 |
| TEAM-1026 | ATCCCATGACCTGTTCCCACCTGAACTCCTCAACGTTATGGCTAT | F-insert2: JBEI-19556 |
| TEAM-1027 | CCTGCAGGTCGACTCTAGAGCTAGTGCGCATTATTGGCTCCC | R-insert2: JBEI-19556 |
| TEAM-1079 | CCTGCAGGTCGACTCTAGAGCTAGTGCGCATTATTGGCTCCC | F-vector: JBEI-19557 |
| TEAM-1080 | TCTCCTTCATCCGAAACGTCCTCTAGAGTCGACCTGCAGGC | R-vector: JBEI-19557 |
| TEAM-1081 | TGTGGGGAGACGTCGAAAAGCCCGGGTACCGAGCT | F-insert1: JBEI-19557 |
| TEAM-1082 | ATTCGAGCTCGGTACCCGGGCTTTTCGACGTCTCCCCAC | R-insert1: JBEI-19557 |
| TEAM-1083 | TCGCCAACTAGGCGCCAAAGCGATCCCACTTCCTGATTTCCC | F-insert2: JBEI-19557 |
| TEAM-1084 | GAAATCAGGAAGTGGGATCGCTTTGGCGCCTAGTTGGC | R-insert2: JBEI-19557 |
