# Supplementary material for: Engineering Corynebacterium glutamicum to produce the biogasoline isopentenol from plant biomass hydrolysates
Source: Biotechnol Biofuels. 2019 Feb 27;12:41. doi: 10.1186/s13068-019-1381-3 (PMC6391826; doi:10.1186/s13068-019-1381-3)
Supplement: Supplementary file 8 — Additional file 8. Additional Methods. Determination of sugars, organic acids and monomeric aromatics in the hydrolysate. [file 13068_2019_1381_MOESM8_ESM.docx]

**Engineering *Corynebacterium glutamicum* to produce isopentenol from plant biomass hydrolysates.**

**Sasaki and Eng et al.**

**Supplementary Files.**

1. **Additional File 1.** Evaluation of Production Condition and *C. glutamicum* Properties as the Isopentenol Production Chassis.
2. **Additional File 2.** Isopentenol Production in *C. glutamicum* Strains Cultivated in Rich vs. Minimal Media.
3. **Additional File 3.** Impact of Initial Glucose and Nitrogen Concentrations On Isopentenol Production in *C. glutamicum.*
4. **Additional File 4.** Analysis of Pathway Protein Abundance vs. Isopentenol Titer in Three Kinds of Media.
5. **Additional File 5.** Determination of sugars and aromatics in hydrolysate by HPLC**.**
6. **Additional File 6.** Strains and plasmids used in this Study.
7. **Additional File 7.** Strain genotyping primers.
8. **Additional File 8.** Supplementary Methods: Determination of sugars, organic acids and monomeric aromatics in the hydrolysate.

**Additional Methods:**

**Determination of sugars, organic acids and monomeric aromatics in the hydrolysate.**

Sugars and organic acids were quantified by HPLC using an Agilent Technologies 1200 series instrument equipped with an Aminex HPX-87H column (BioRad Laboratories, USA) and a refractive index detector. 4 mM sulfuric acid was used as mobile phase with a flow rate of 0.6 mL/min and a column temperature of 60°C. To quantify monomeric aromatics, the same instrument equipped with an Eclipse Plus Phenyl-hexyl column (250 mm length, 4.6 mm diameter, 5 µm particle size; Agilent Technologies, USA) kept at 50 ºC, and a diode array detector were used. The mobile phase was composed of 10 mM ammonium acetate in water (solvent A) and 10 mM ammonium acetate in acetonitrile 90% (solvent B), prepared from a stock solution of 100 mM ammonium acetate and 0.7% formic acid in water. The following mobile phase gradient profile was used: 30% B (0 min; 0.5 mL/min), 80% B (12 min; 0.5 mL/min), 100% B (12.1 min; 0.5 mL/min), 100% B (12.6 min; 1 mL/min), 30% B (12.8 min; 1 mL/min), 30% B (15.6 min; 1 mL/min). Prior to analysis, samples were filtered using 0.45 µm centrifuge filters and 5 µL sample injection volumes were used in all cases. The resulting peak areas were compared to calibration curves made with authentic standards.
